# Supplementary figures and images for: In vitro effect of resin infiltrant on resistance of sound enamel surfaces in permanent teeth to demineralization
Source: PeerJ. 2021 Aug 13;9:e12008. doi: 10.7717/peerj.12008 (PMC8759355; doi:10.7717/peerj.12008)

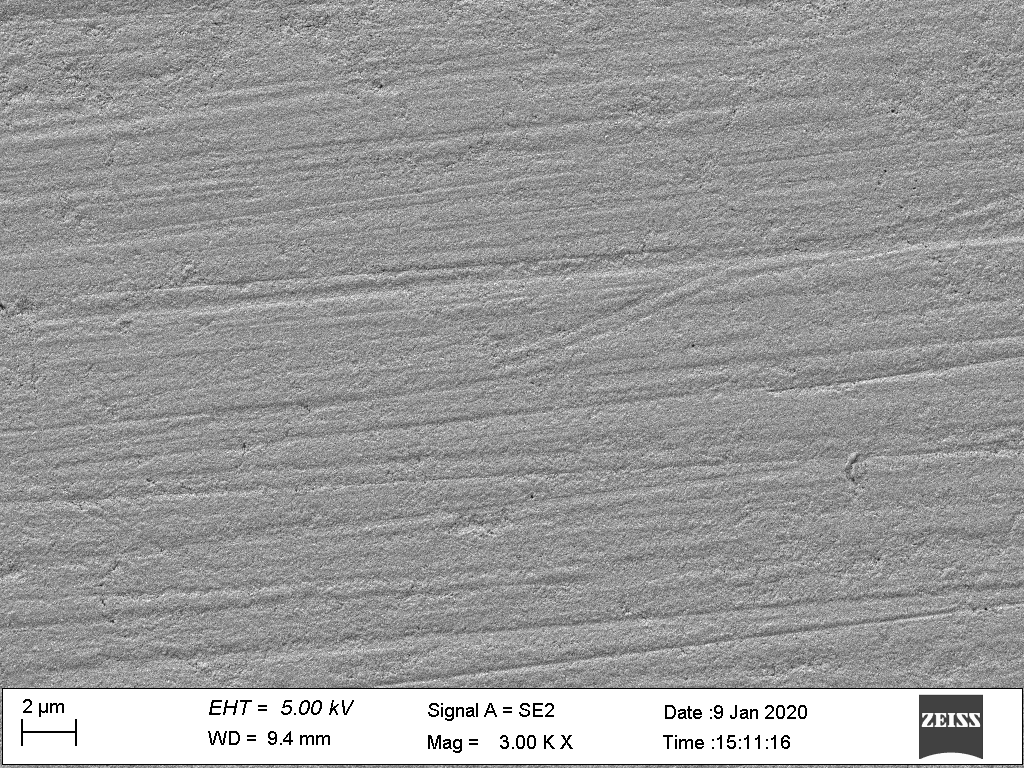

Supplement: Supplemental Information 2 [file peerj-09-12008-s002.zip › Scanning electron microscopy/Fig. 3A/1-3000×.png]

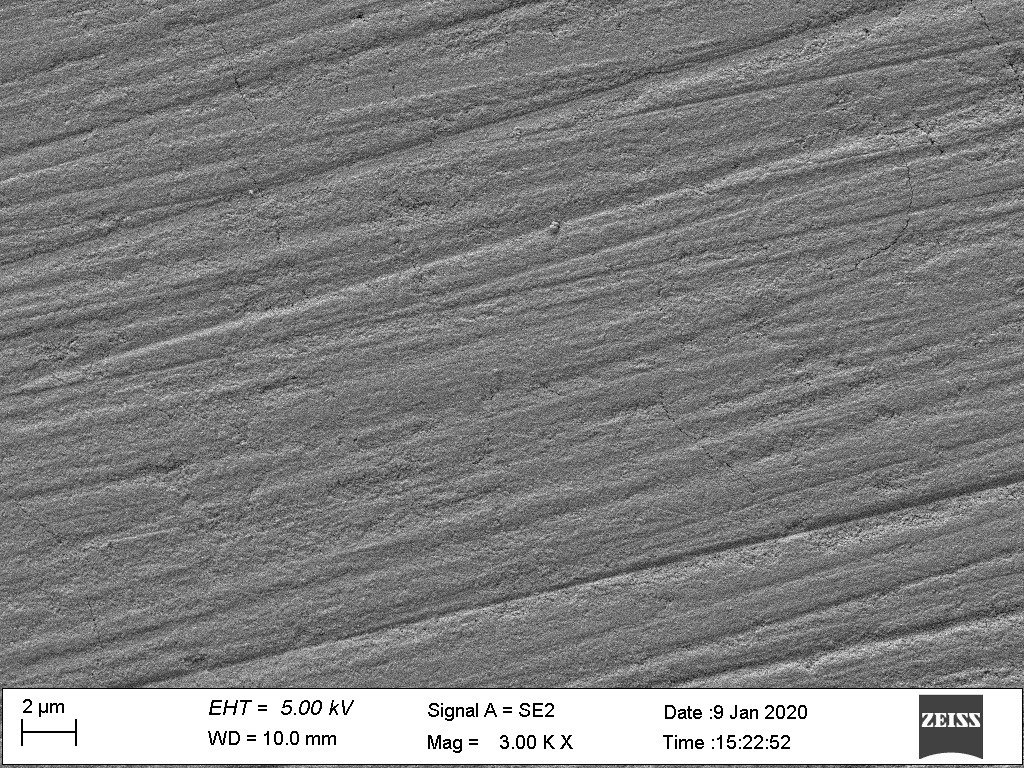

Supplement: Supplemental Information 2 [file peerj-09-12008-s002.zip › Scanning electron microscopy/Fig. 3A/2-3000×.png]

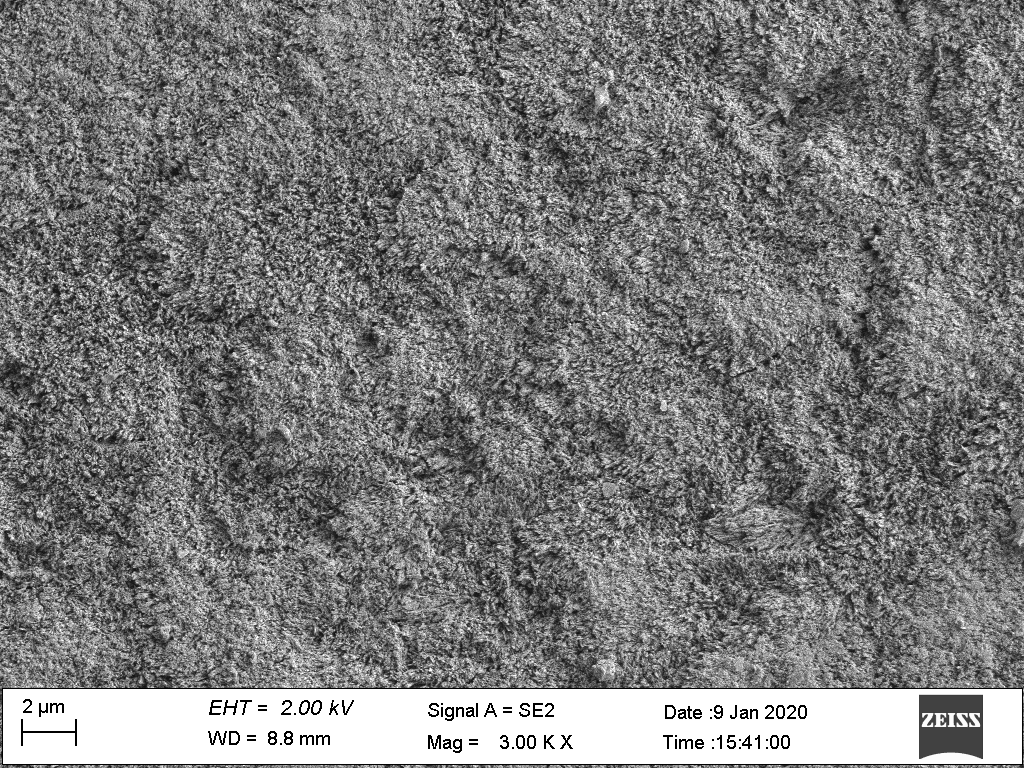

Supplement: Supplemental Information 2 [file peerj-09-12008-s002.zip › Scanning electron microscopy/Fig. 3B/1-3000×.png]

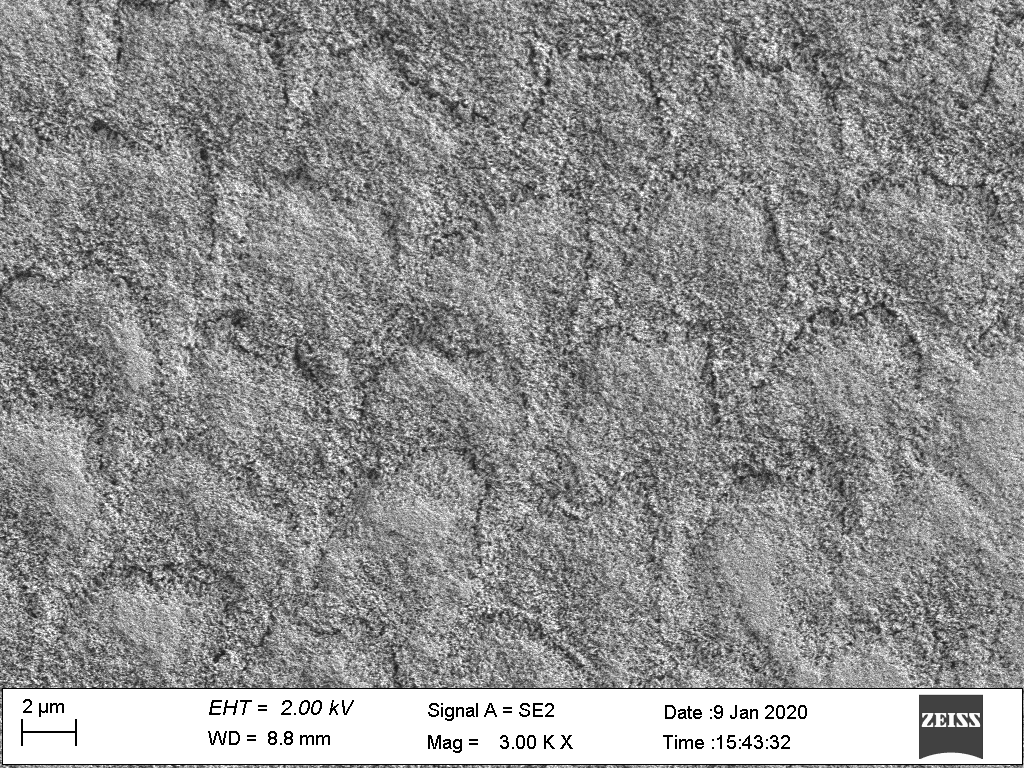

Supplement: Supplemental Information 2 [file peerj-09-12008-s002.zip › Scanning electron microscopy/Fig. 3B/2-3000×.png]

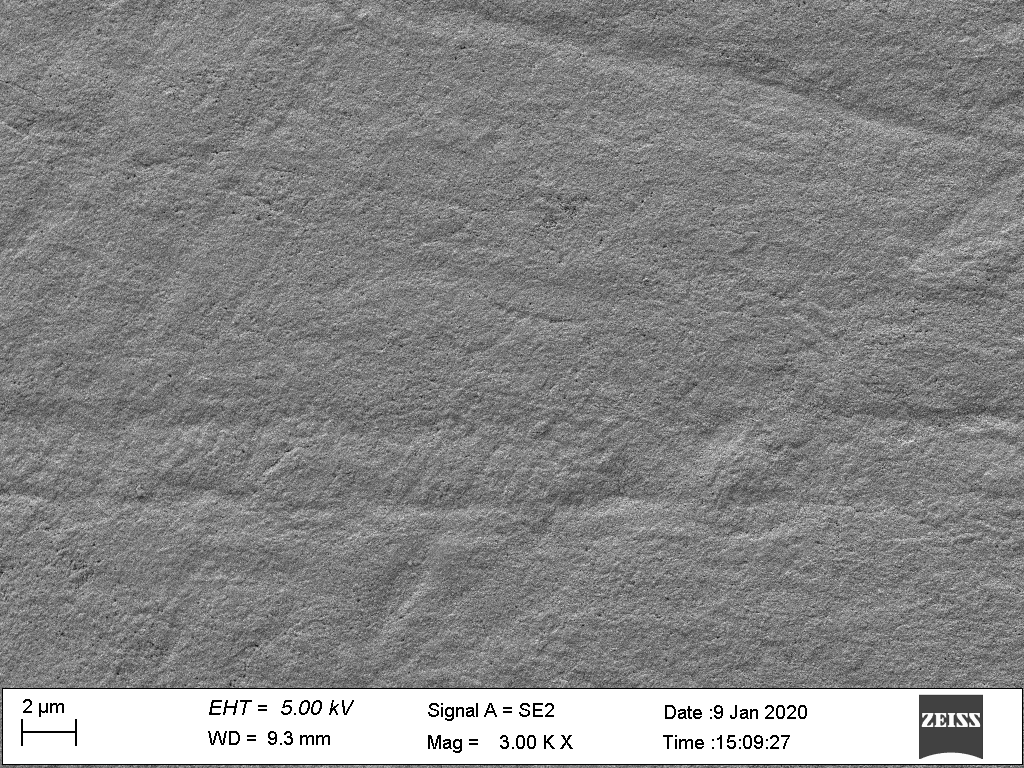

Supplement: Supplemental Information 2 [file peerj-09-12008-s002.zip › Scanning electron microscopy/Fig. 3C/1-3000×.png]

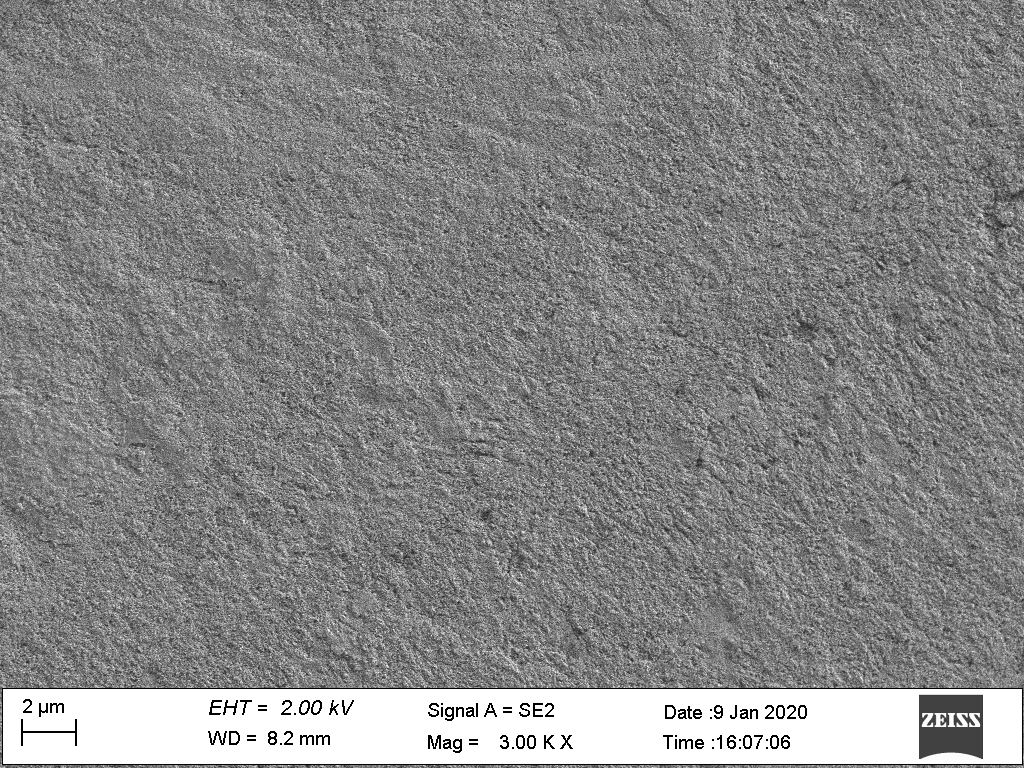

Supplement: Supplemental Information 2 [file peerj-09-12008-s002.zip › Scanning electron microscopy/Fig. 3C/2-3000×.png]

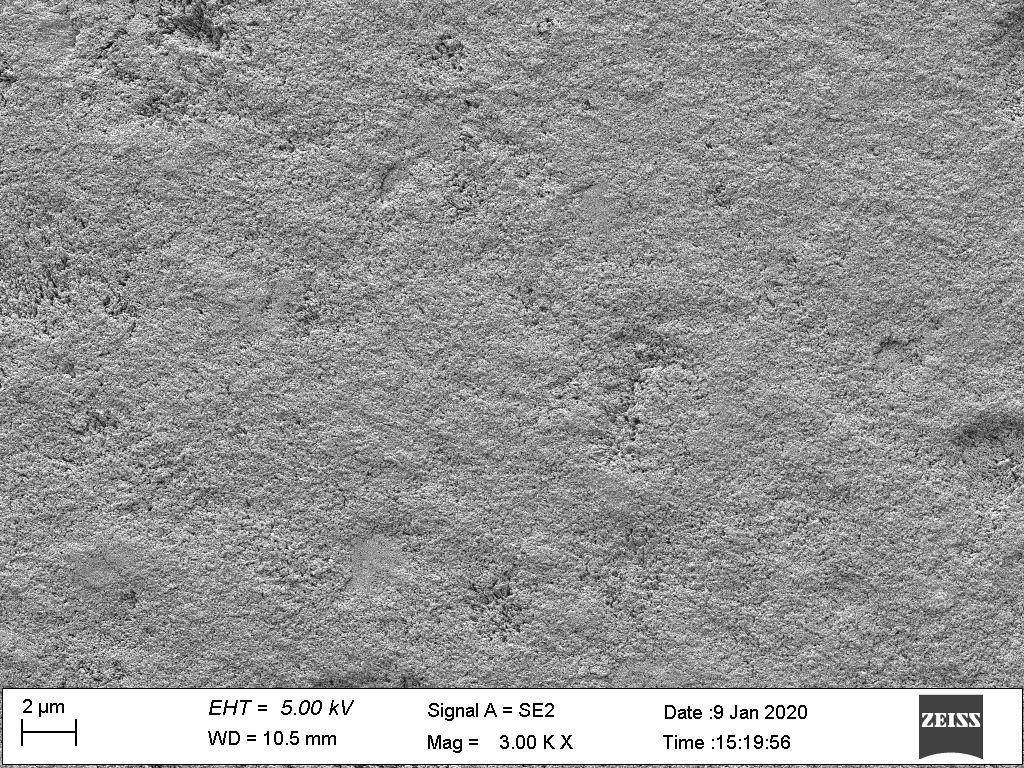

Supplement: Supplemental Information 2 [file peerj-09-12008-s002.zip › Scanning electron microscopy/Fig. 3C/3-3000×.png]

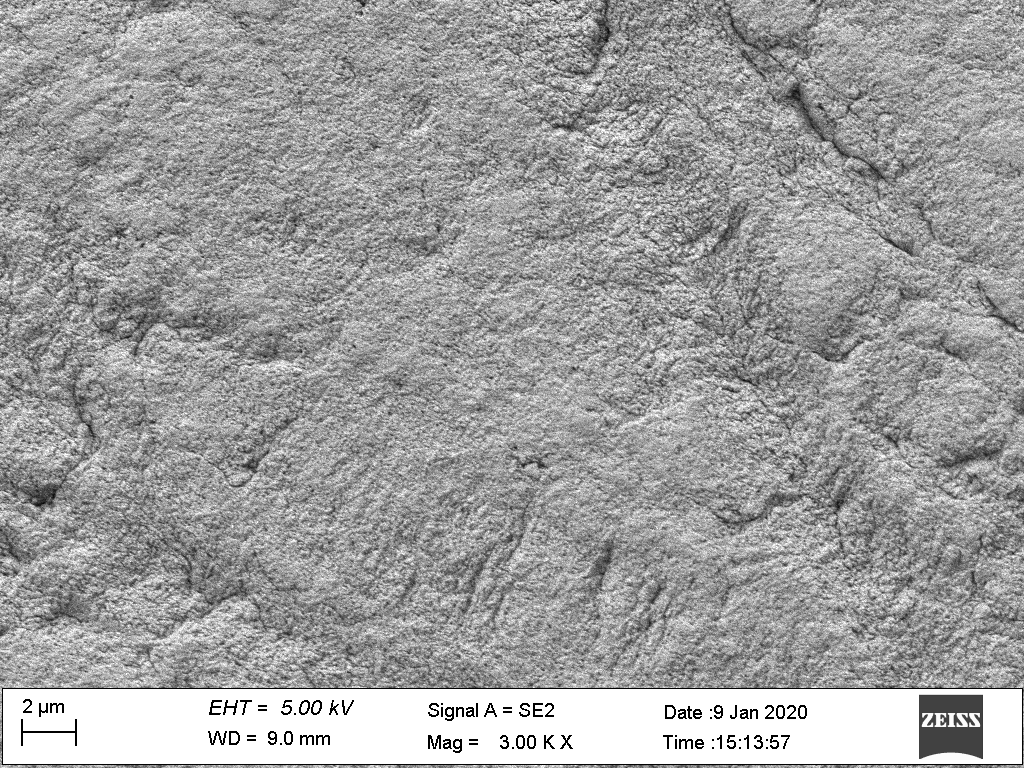

Supplement: Supplemental Information 2 [file peerj-09-12008-s002.zip › Scanning electron microscopy/Fig. 3D/1-3000×.png]

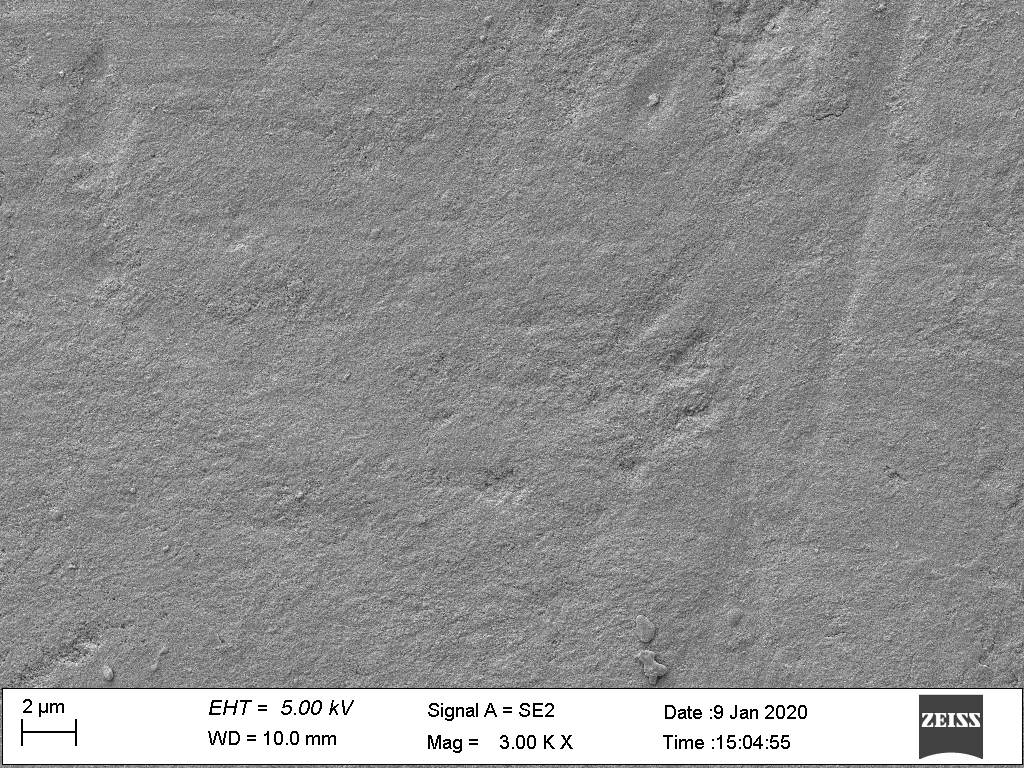

Supplement: Supplemental Information 2 [file peerj-09-12008-s002.zip › Scanning electron microscopy/Fig. 3D/2-3000×.png]

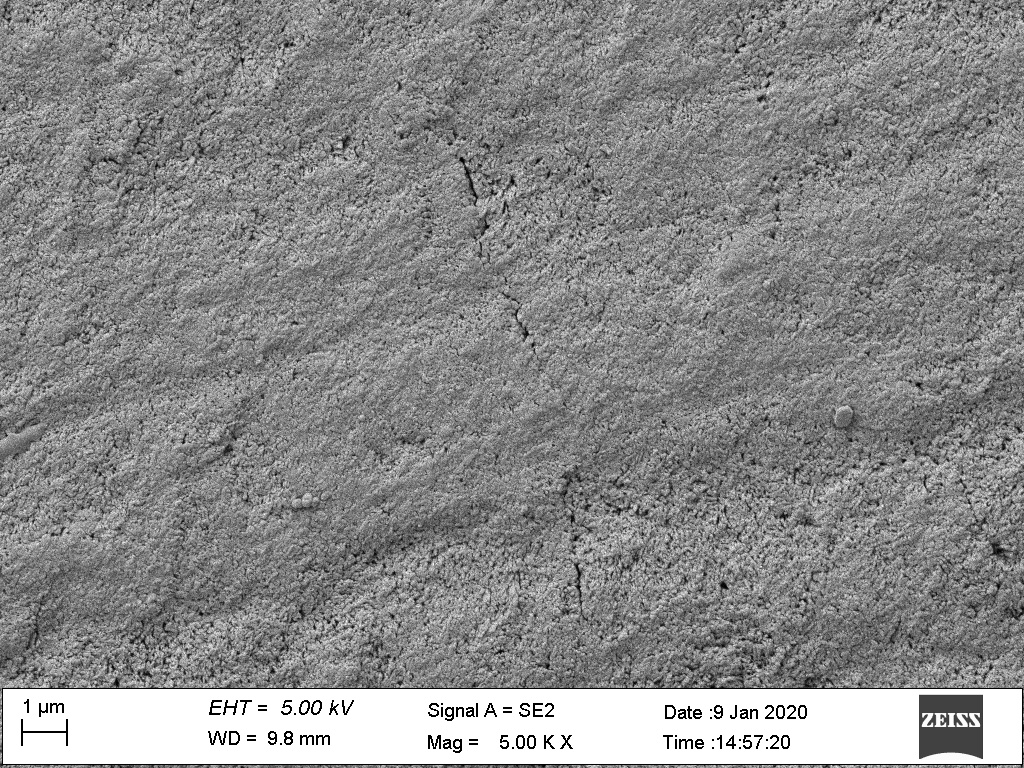

Supplement: Supplemental Information 2 [file peerj-09-12008-s002.zip › Scanning electron microscopy/Fig. 3D/3-3000×.png]

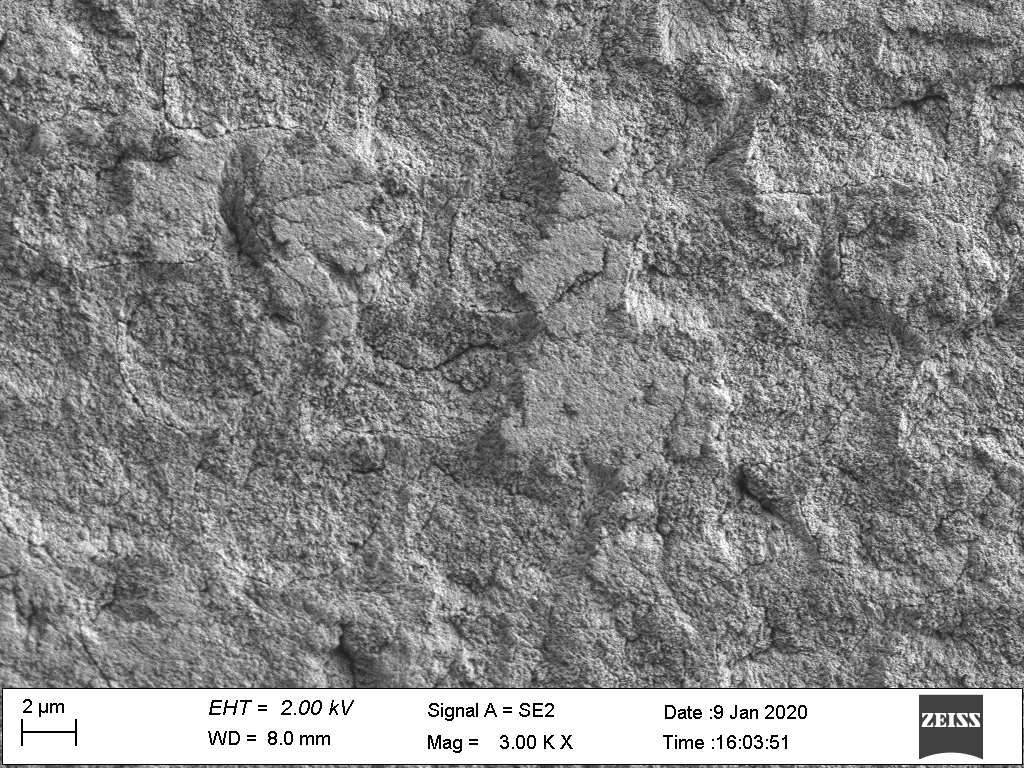

Supplement: Supplemental Information 2 [file peerj-09-12008-s002.zip › Scanning electron microscopy/Fig. 3E/1-3000×.png]

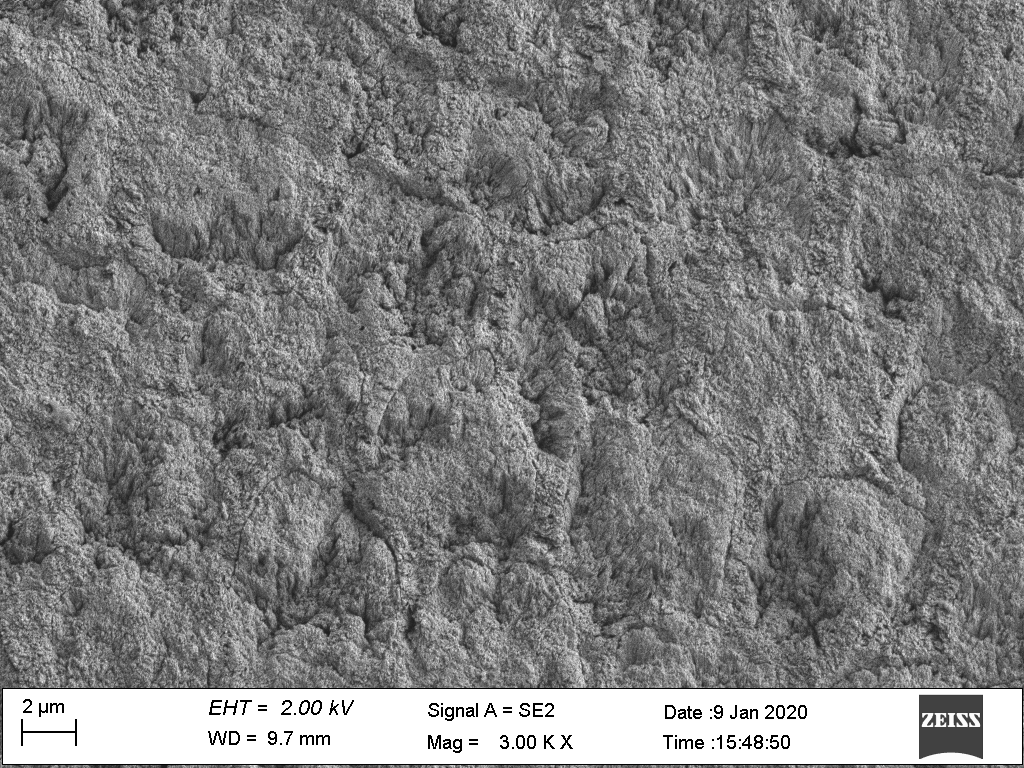

Supplement: Supplemental Information 2 [file peerj-09-12008-s002.zip › Scanning electron microscopy/Fig. 3E/2-3000×.png]

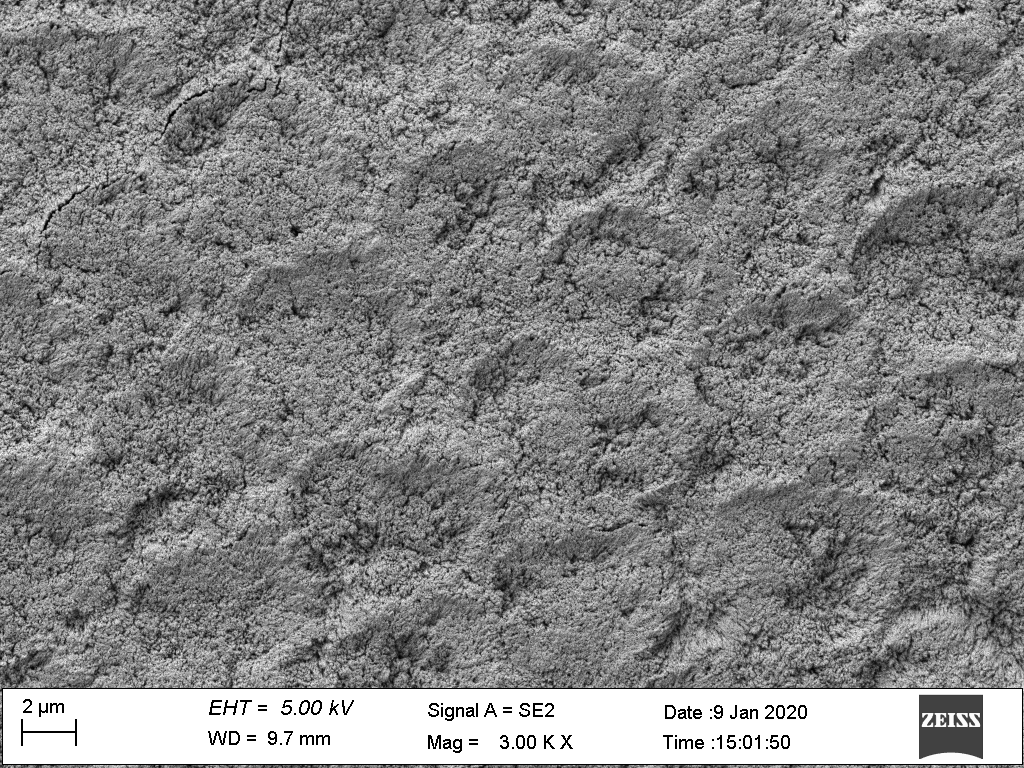

Supplement: Supplemental Information 2 [file peerj-09-12008-s002.zip › Scanning electron microscopy/Fig. 3E/3-3000×.png]

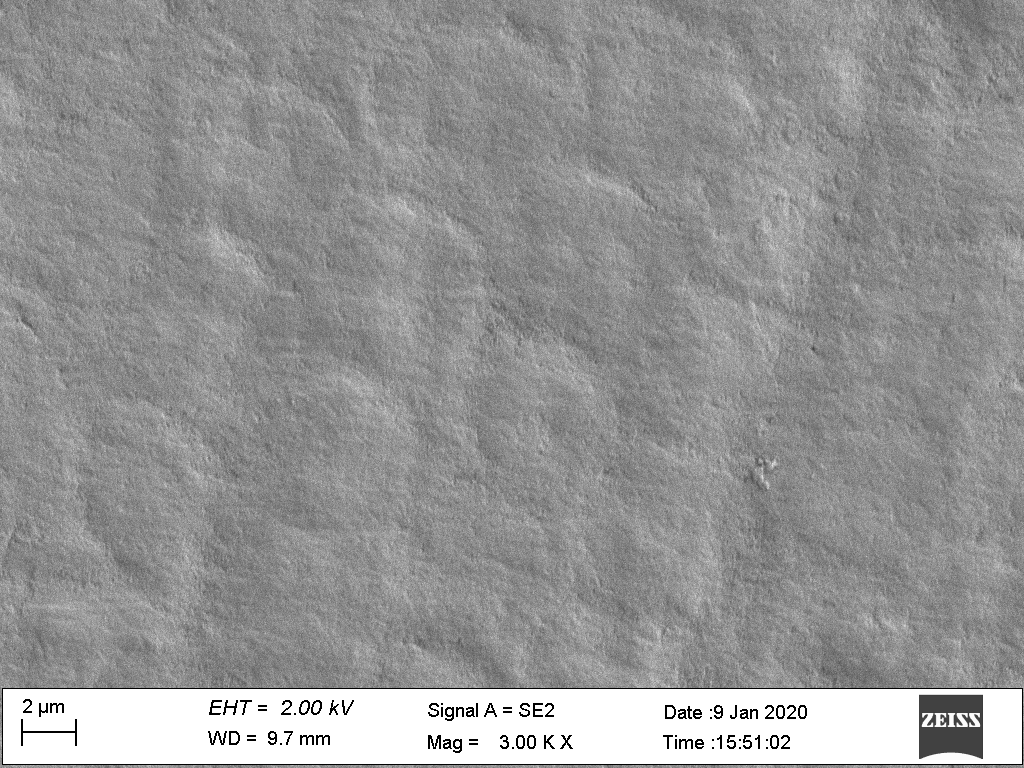

Supplement: Supplemental Information 2 [file peerj-09-12008-s002.zip › Scanning electron microscopy/Fig. 3F/1-3000×.png]

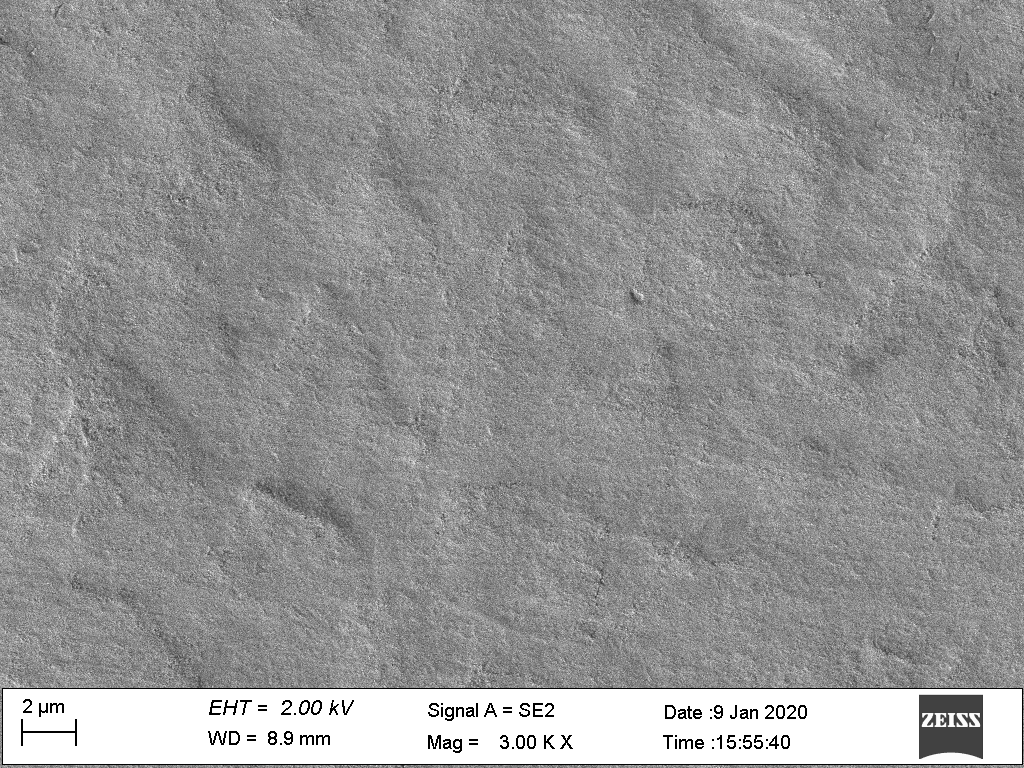

Supplement: Supplemental Information 2 [file peerj-09-12008-s002.zip › Scanning electron microscopy/Fig. 3F/2-3000×.png]

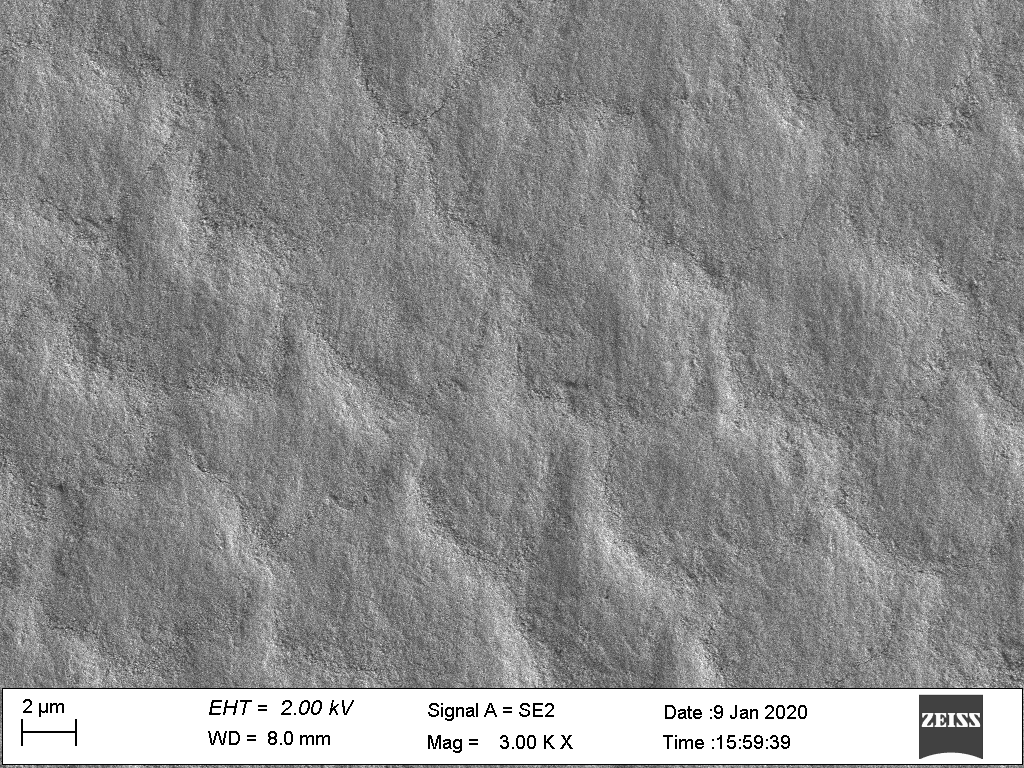

Supplement: Supplemental Information 2 [file peerj-09-12008-s002.zip › Scanning electron microscopy/Fig. 3F/3-3000×.png]

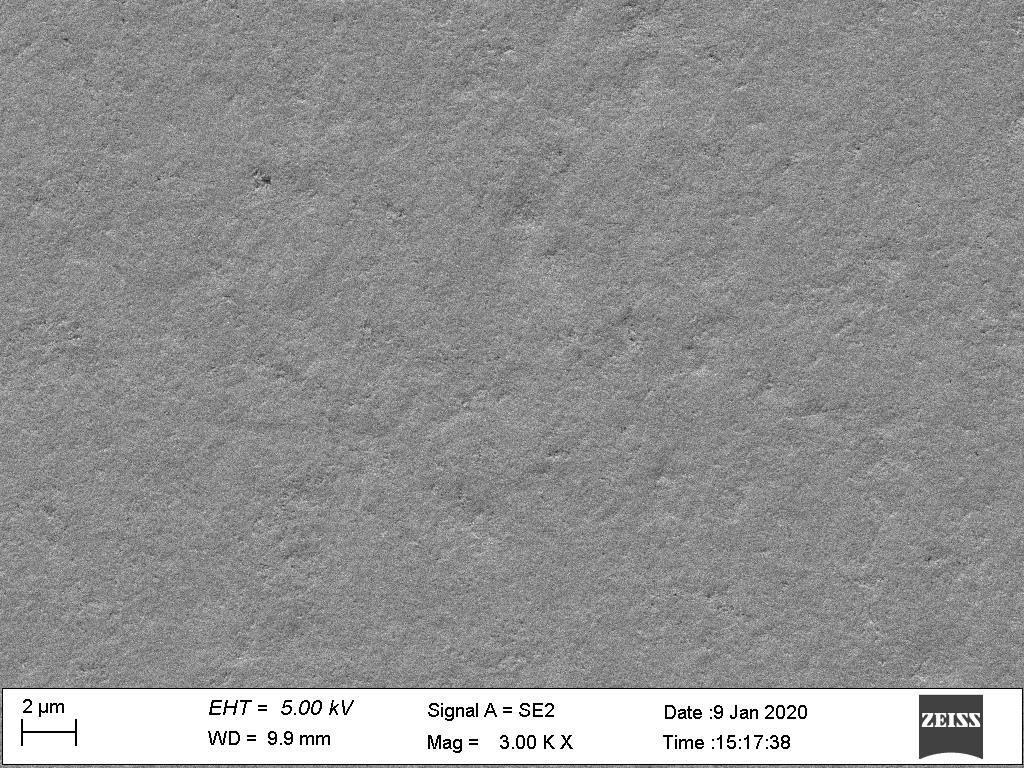

Supplement: Supplemental Information 2 [file peerj-09-12008-s002.zip › Scanning electron microscopy/Fig. 3G/1-3000×.png]

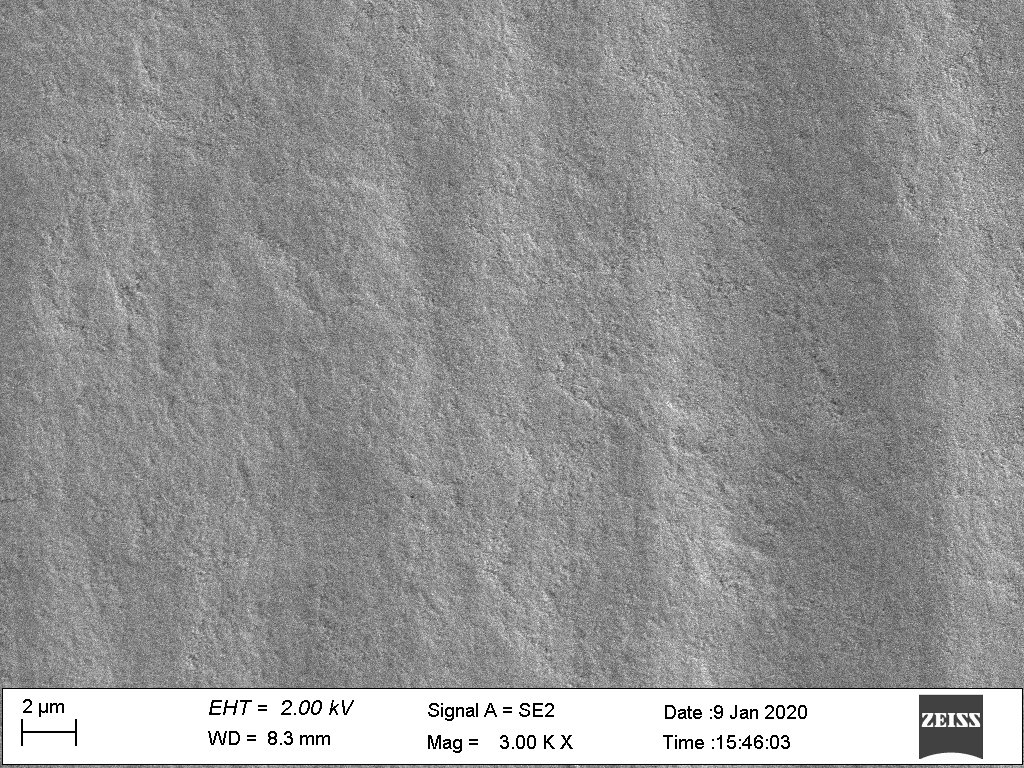

Supplement: Supplemental Information 2 [file peerj-09-12008-s002.zip › Scanning electron microscopy/Fig. 3G/2-3000×.png]

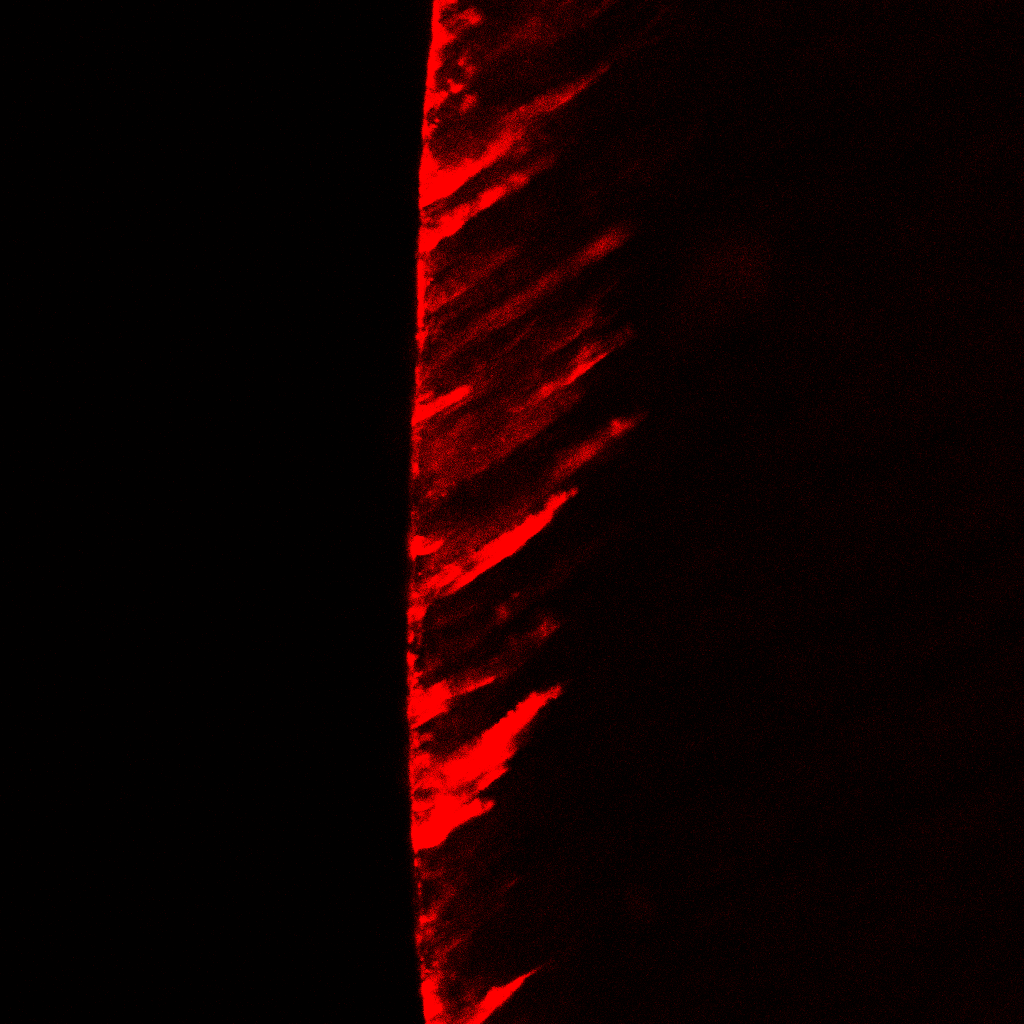

Supplement: Supplemental Information 3 [file peerj-09-12008-s003.zip › experimental group stained with 0.1% Rhodamine B/10.png]

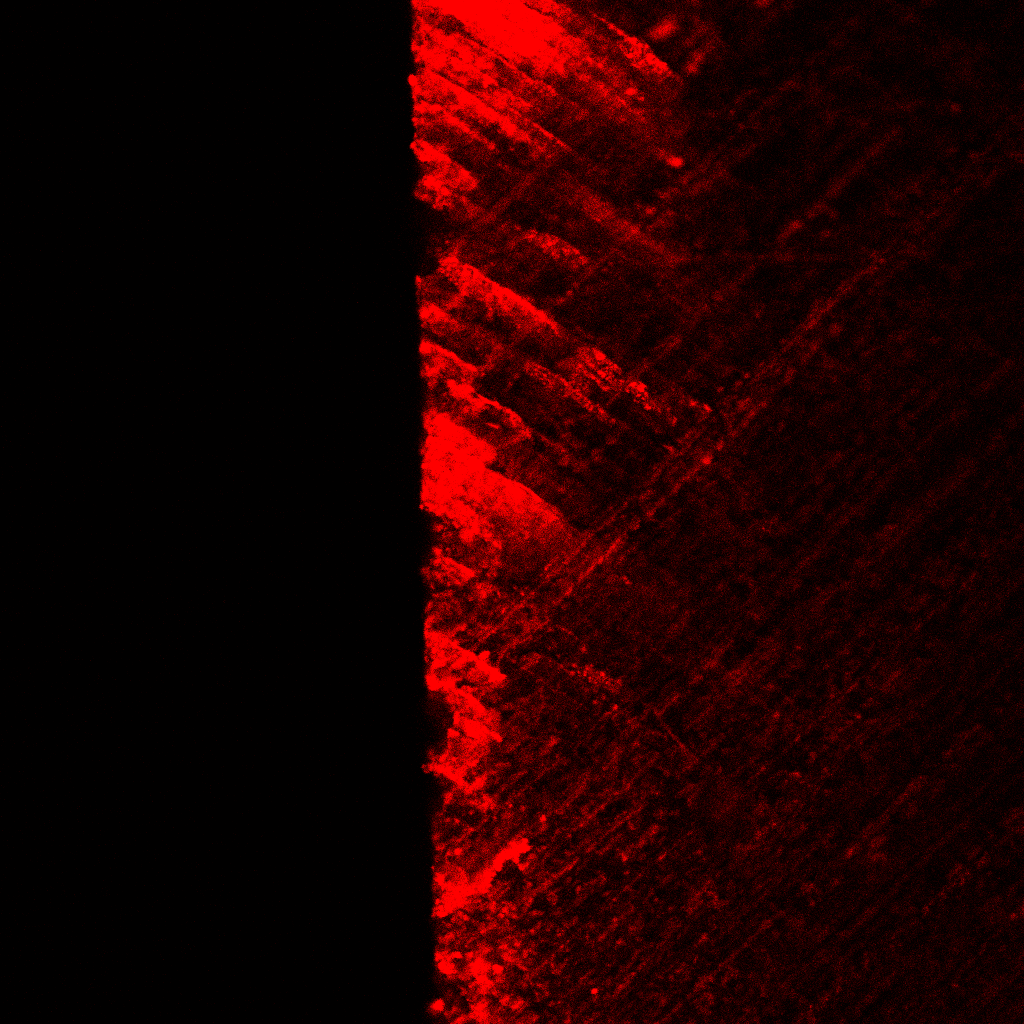

Supplement: Supplemental Information 3 [file peerj-09-12008-s003.zip › experimental group stained with 0.1% Rhodamine B/2.png]

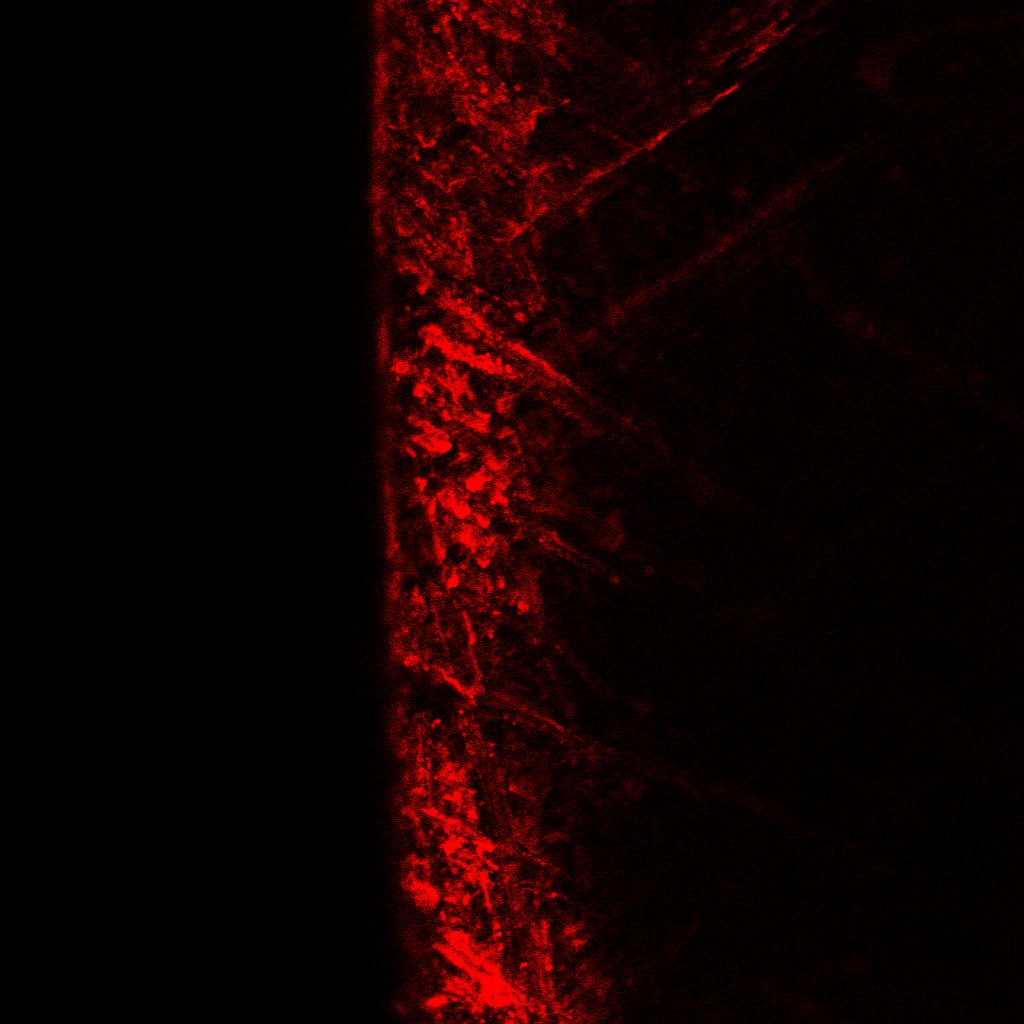

Supplement: Supplemental Information 3 [file peerj-09-12008-s003.zip › experimental group stained with 0.1% Rhodamine B/24.png]

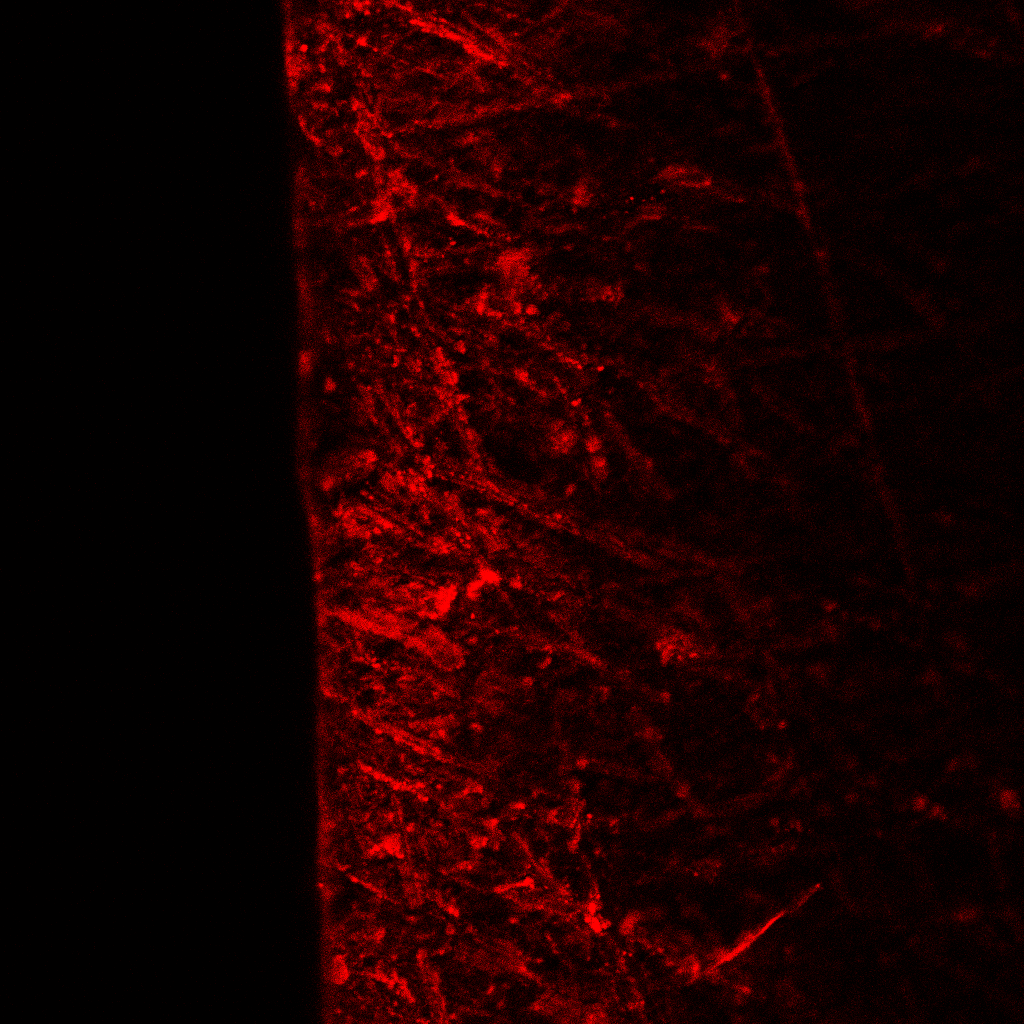

Supplement: Supplemental Information 3 [file peerj-09-12008-s003.zip › experimental group stained with 0.1% Rhodamine B/25.png]

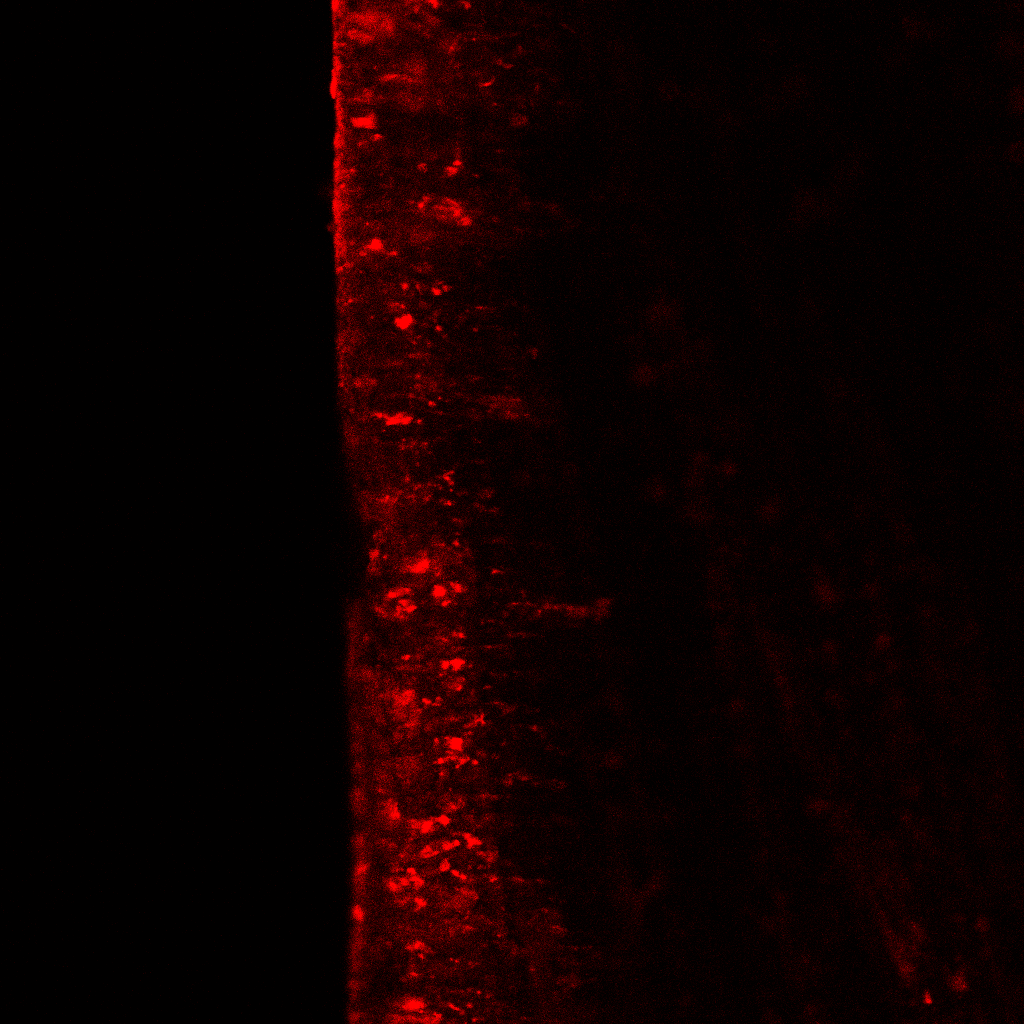

Supplement: Supplemental Information 3 [file peerj-09-12008-s003.zip › experimental group stained with 0.1% Rhodamine B/27.png]

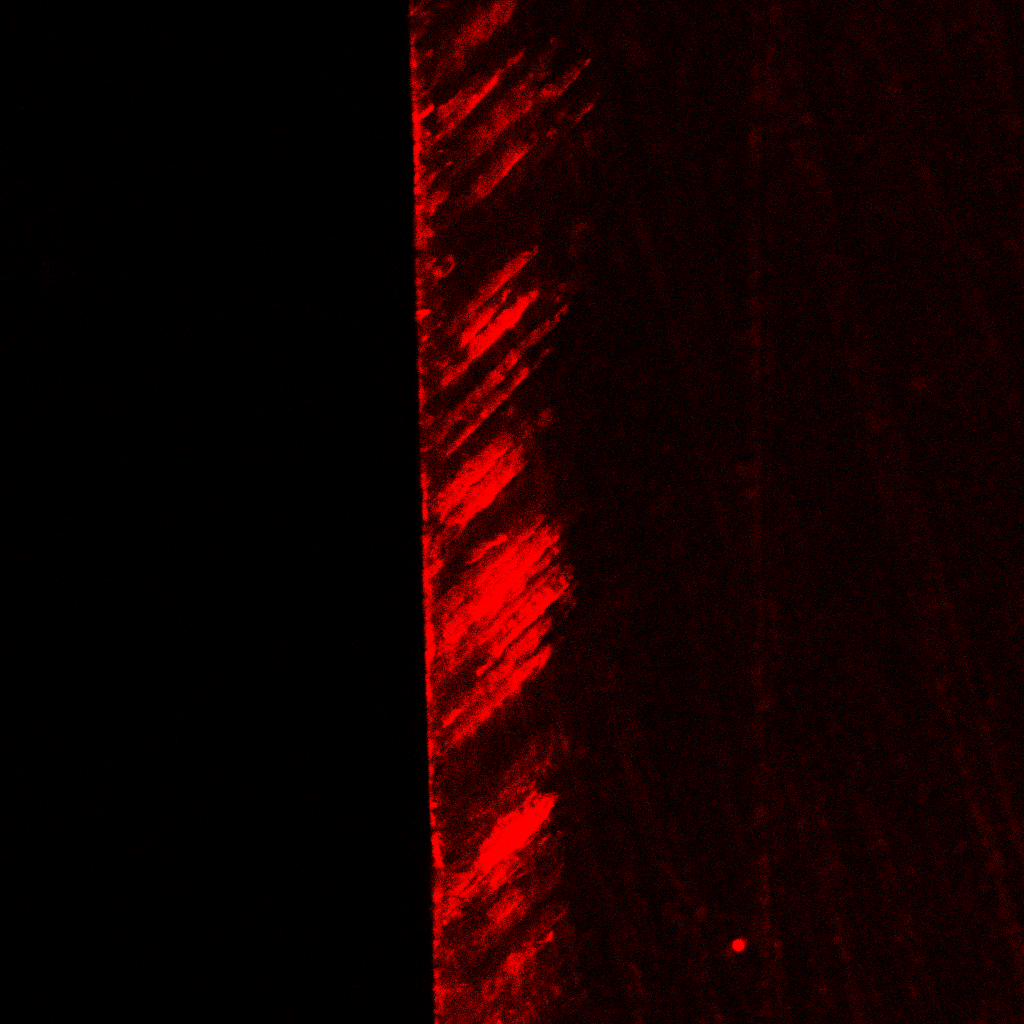

Supplement: Supplemental Information 3 [file peerj-09-12008-s003.zip › experimental group stained with 0.1% Rhodamine B/3.png]

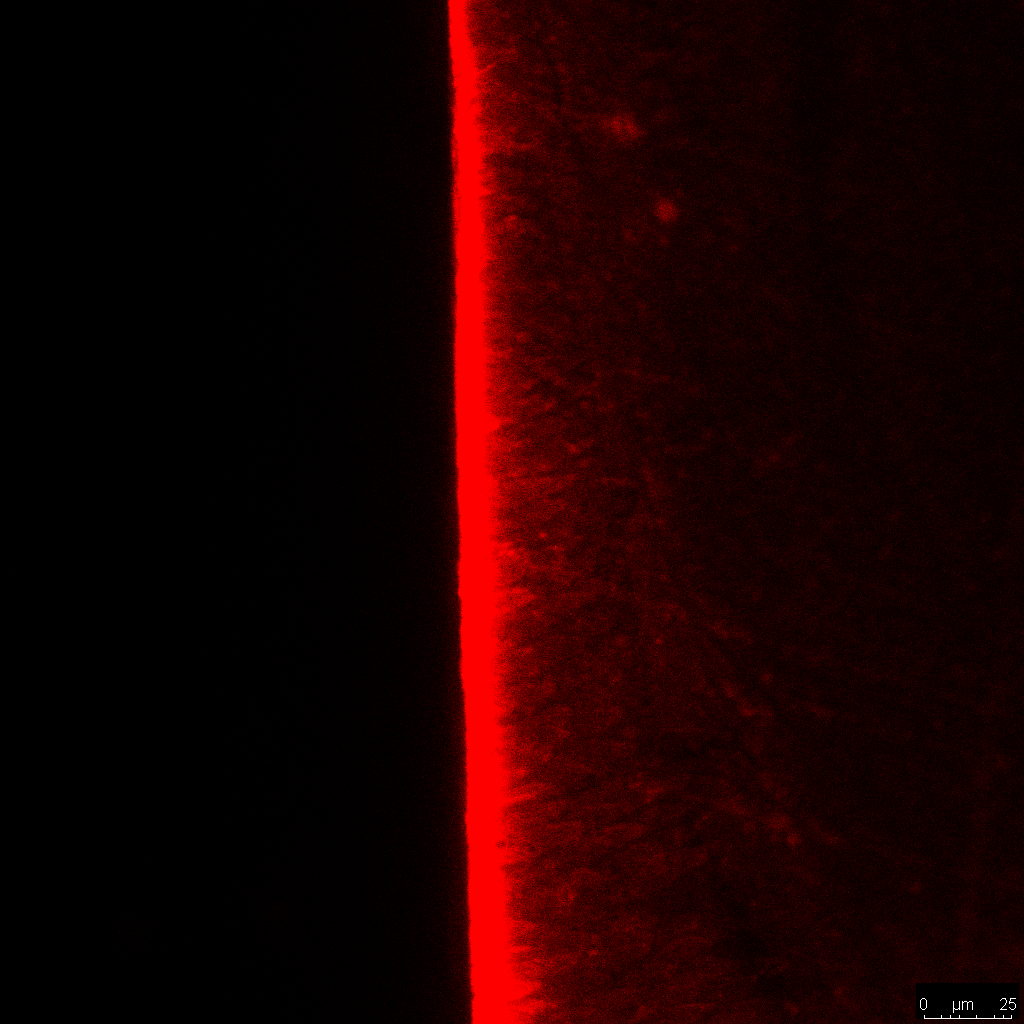

Supplement: Supplemental Information 3 [file peerj-09-12008-s003.zip › experimental group stained with 0.1% Rhodamine B/41.png]

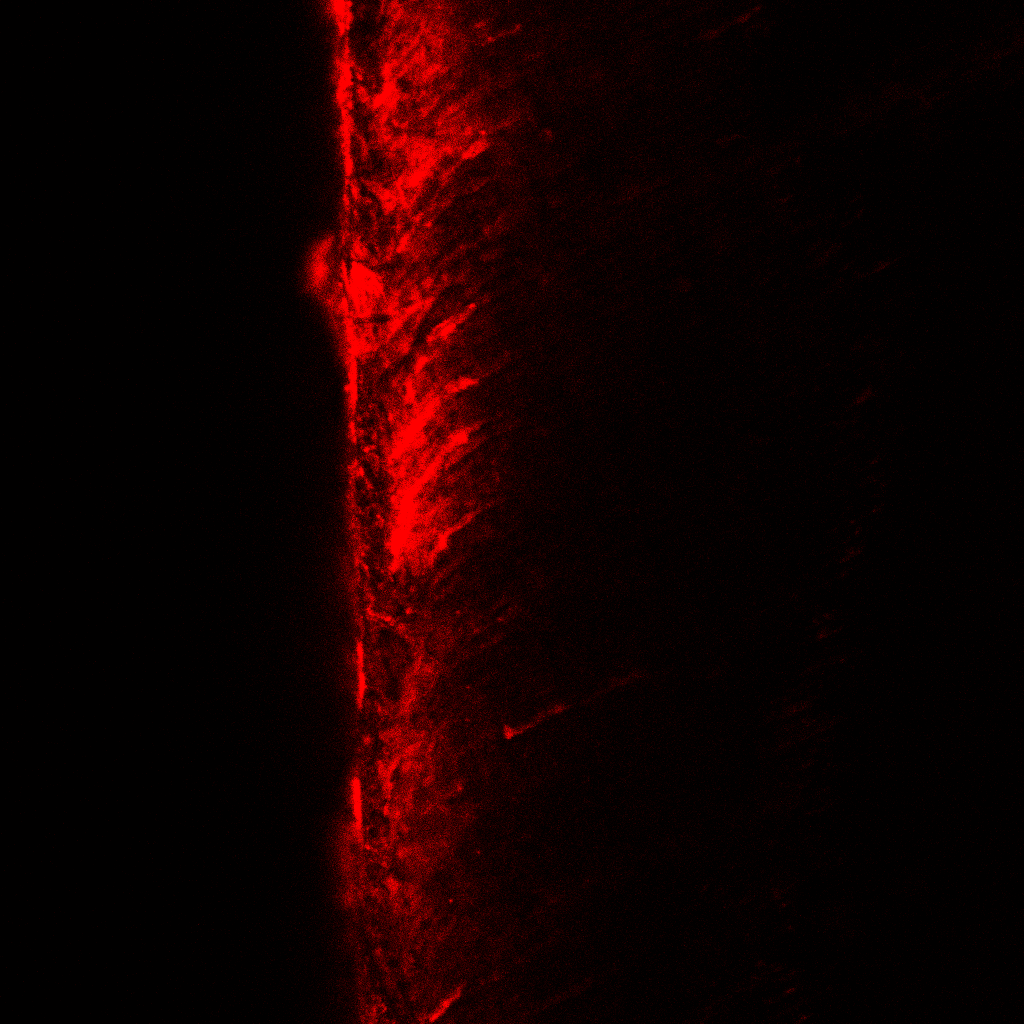

Supplement: Supplemental Information 3 [file peerj-09-12008-s003.zip › experimental group stained with 0.1% Rhodamine B/54.png]

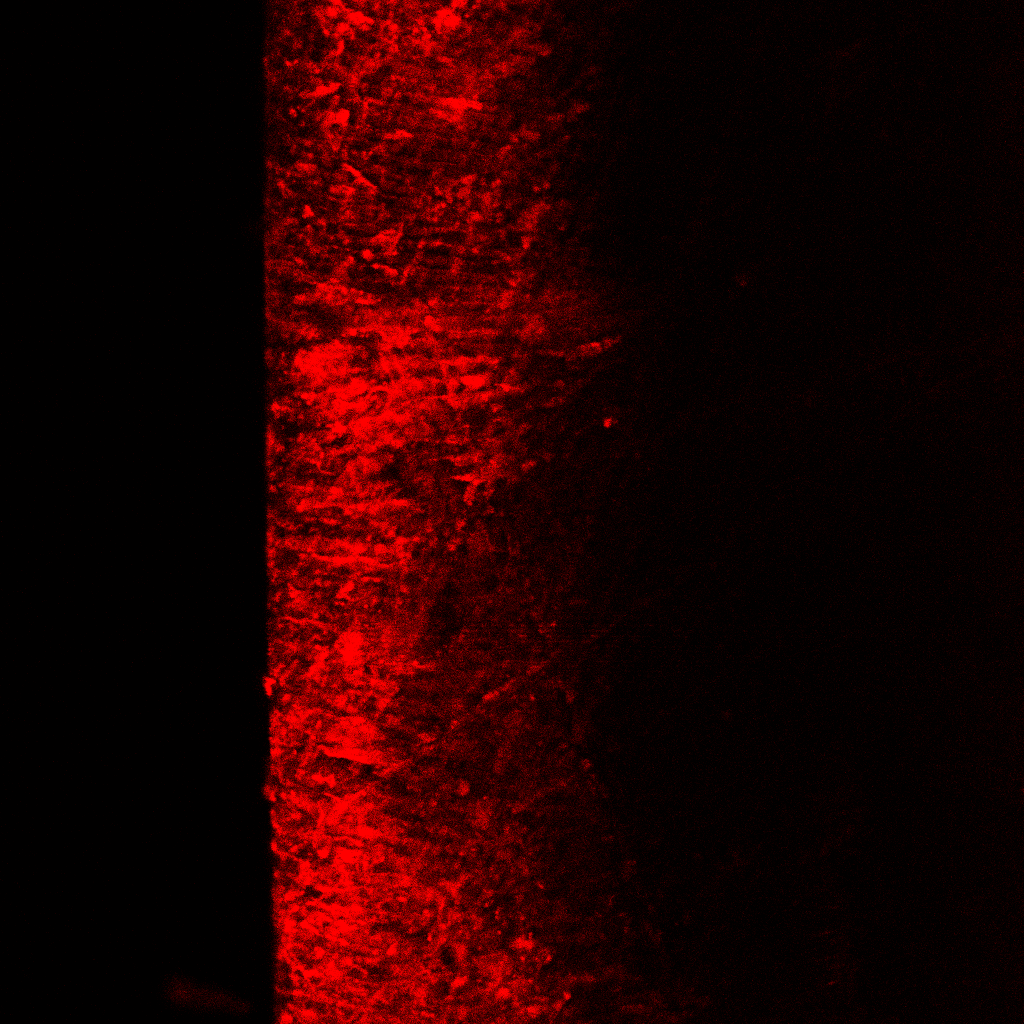

Supplement: Supplemental Information 3 [file peerj-09-12008-s003.zip › experimental group stained with 0.1% Rhodamine B/61.png]

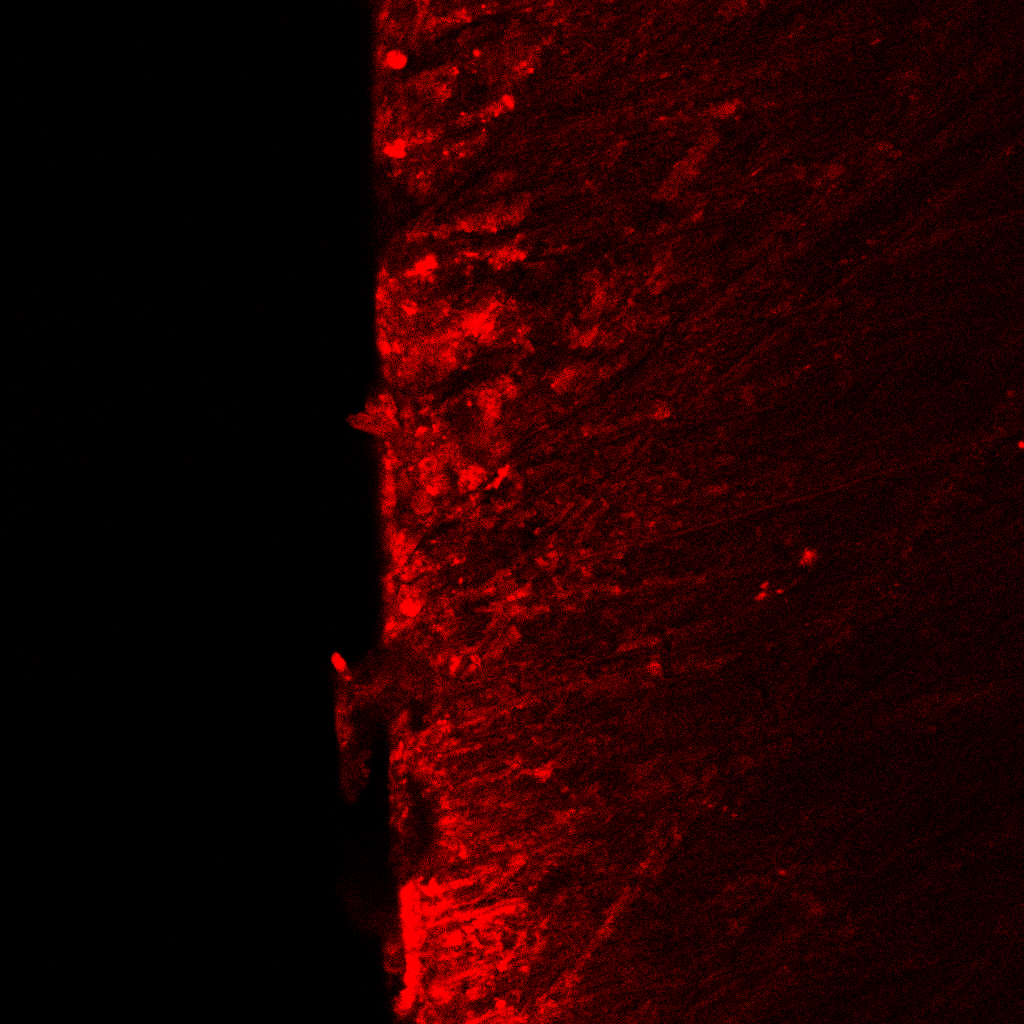

Supplement: Supplemental Information 3 [file peerj-09-12008-s003.zip › experimental group stained with 0.1% Rhodamine B/79.png]

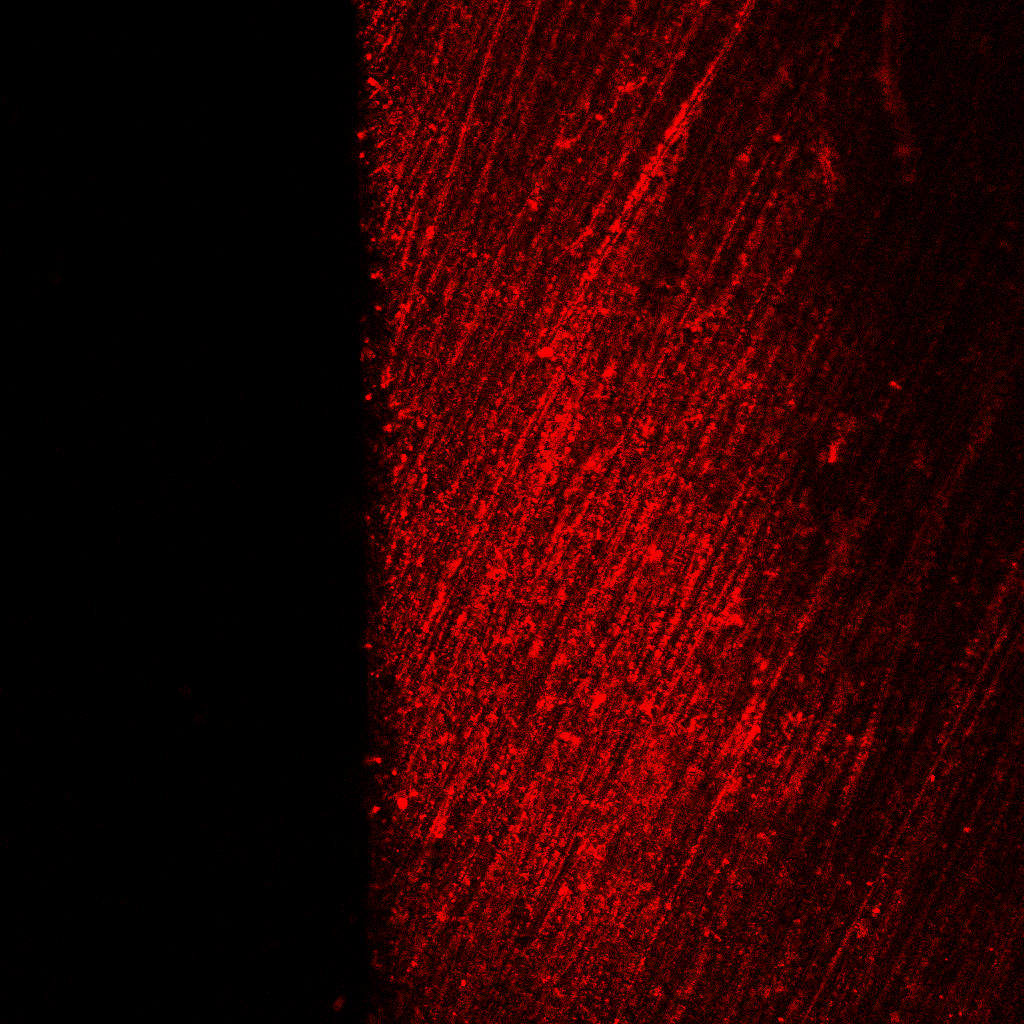

Supplement: Supplemental Information 3 [file peerj-09-12008-s003.zip › control group stained with 0.1% Rhodamine B/1.png]

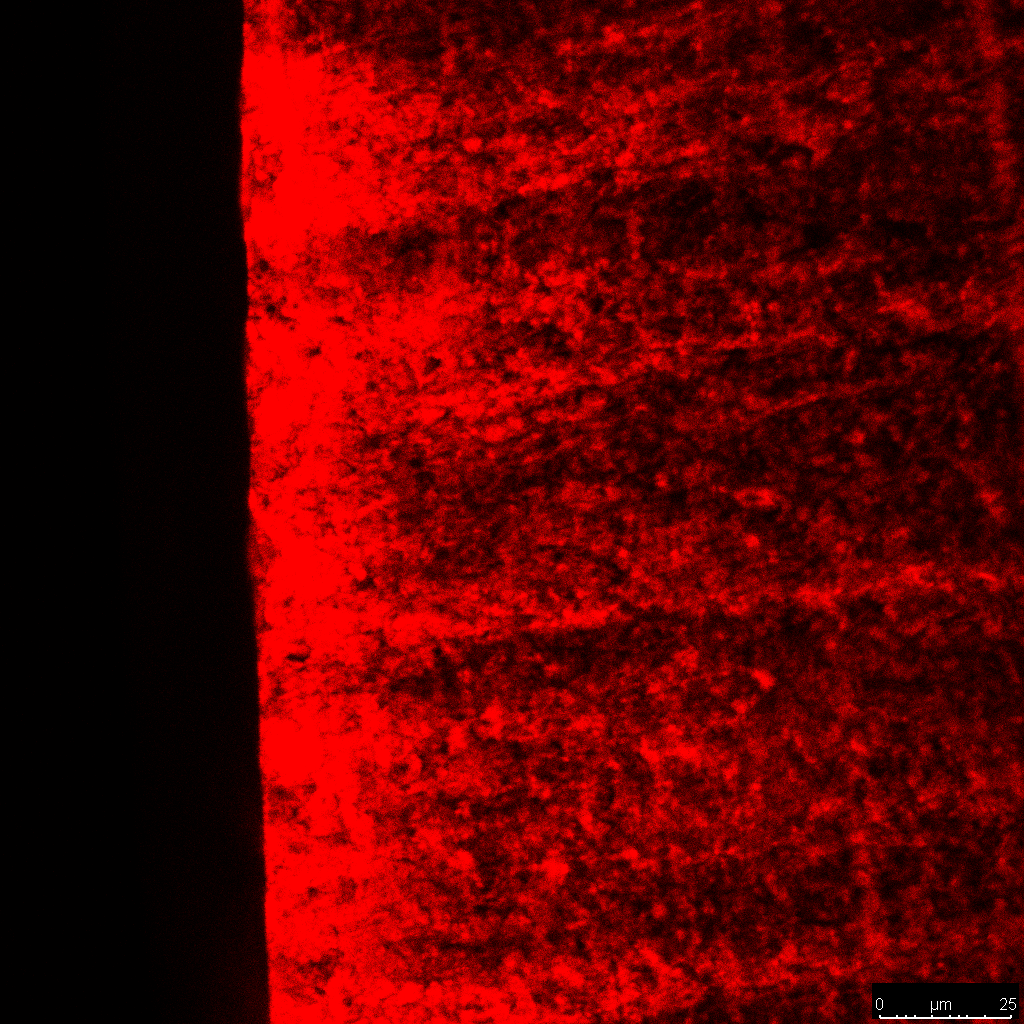

Supplement: Supplemental Information 3 [file peerj-09-12008-s003.zip › control group stained with 0.1% Rhodamine B/21.png]

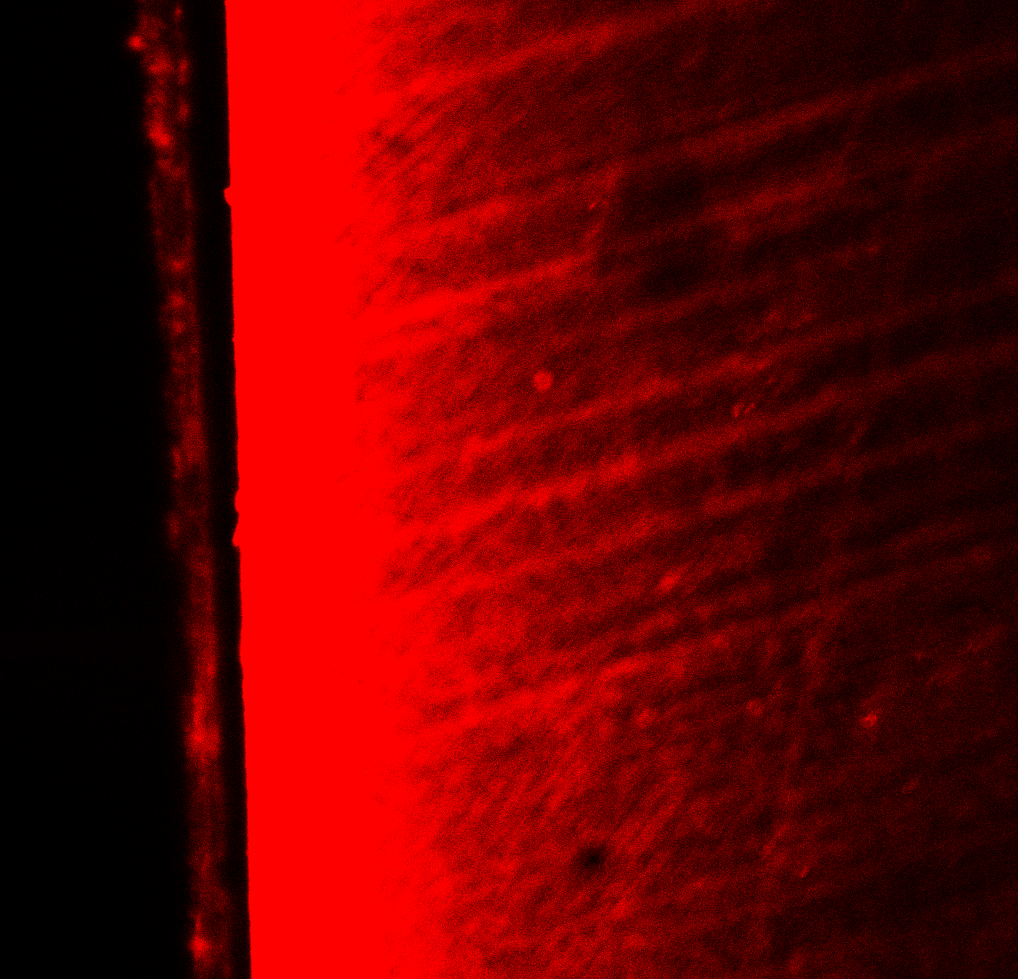

Supplement: Supplemental Information 3 [file peerj-09-12008-s003.zip › control group stained with 0.1% Rhodamine B/31.png]

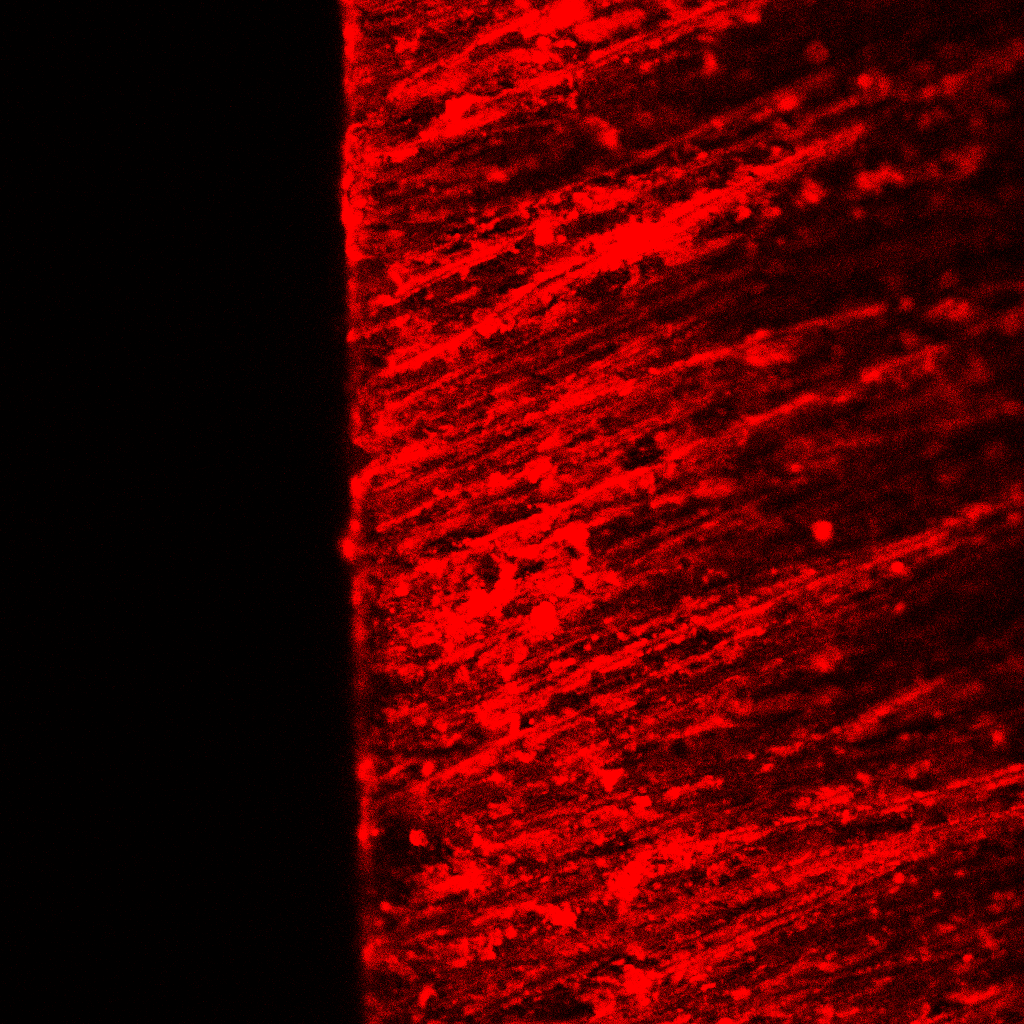

Supplement: Supplemental Information 3 [file peerj-09-12008-s003.zip › control group stained with 0.1% Rhodamine B/32.png]

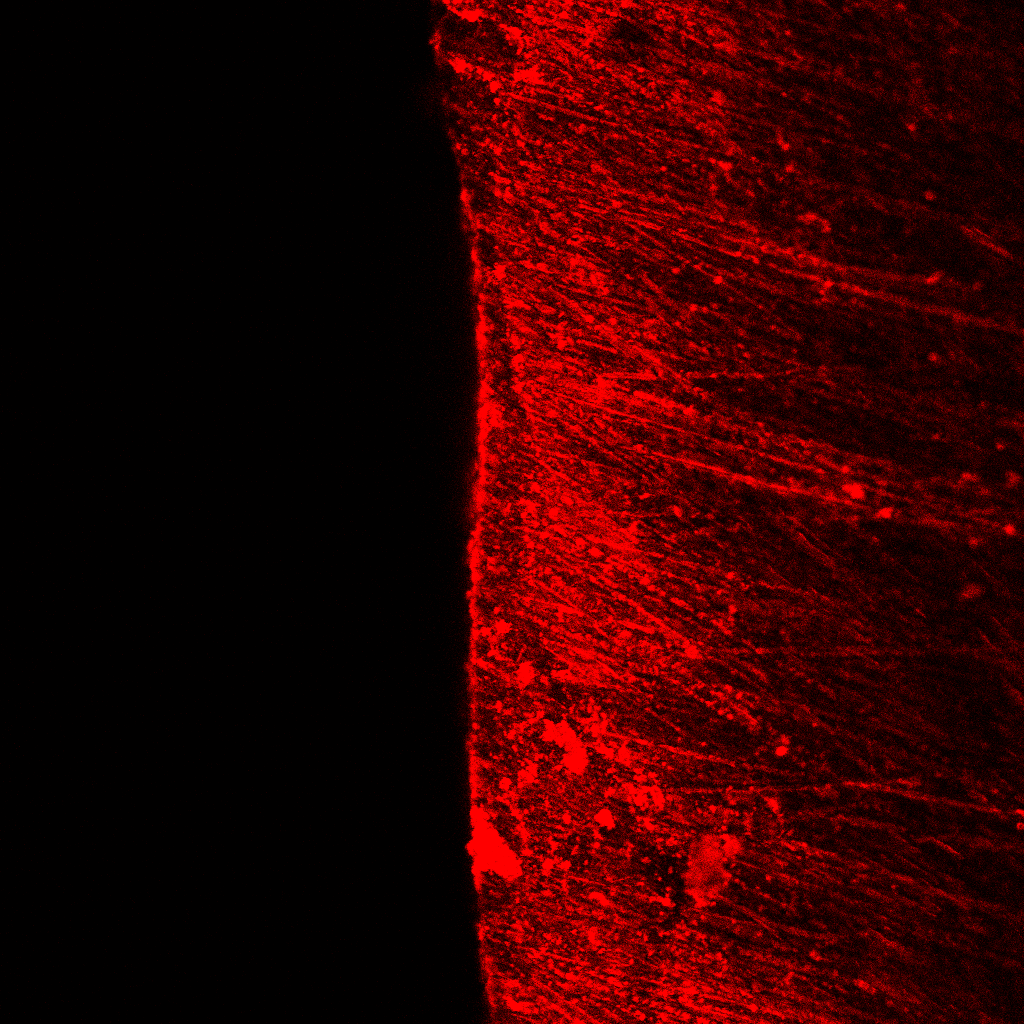

Supplement: Supplemental Information 3 [file peerj-09-12008-s003.zip › control group stained with 0.1% Rhodamine B/33.png]

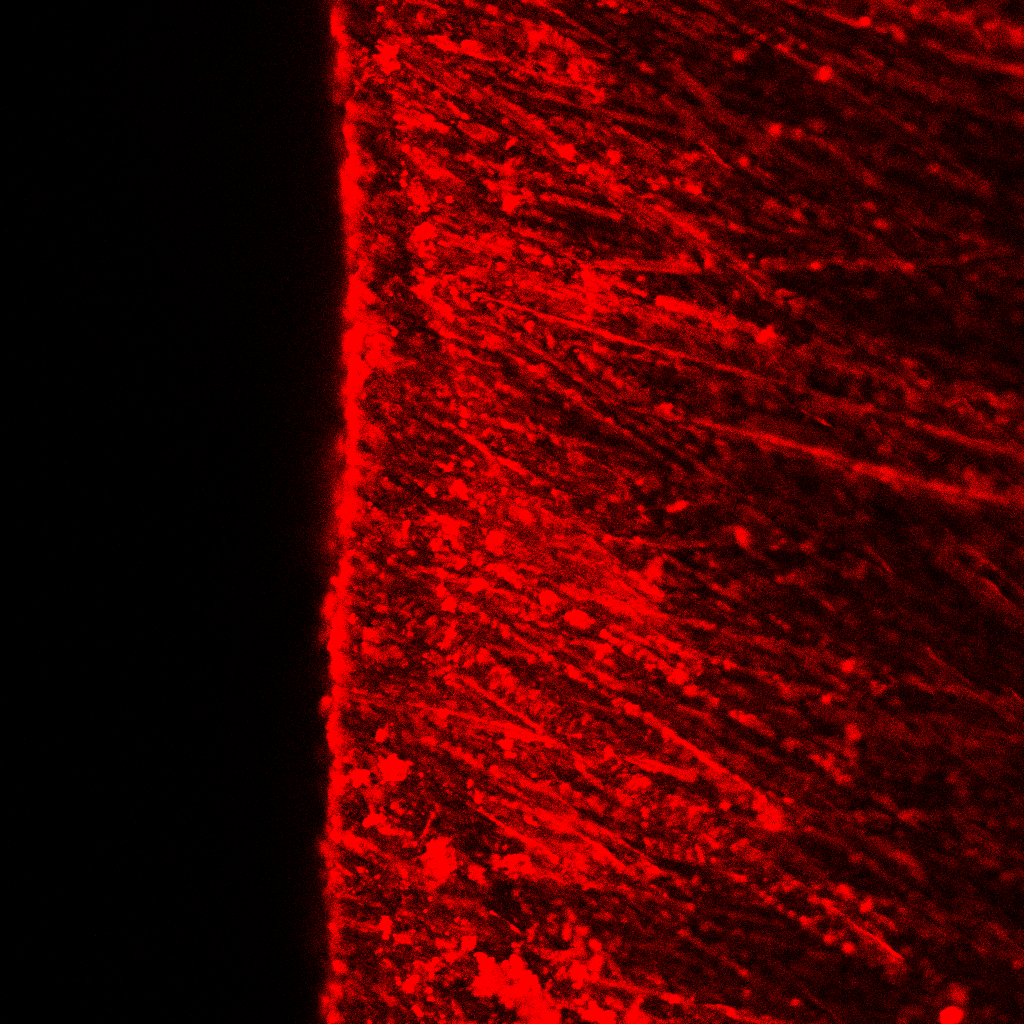

Supplement: Supplemental Information 3 [file peerj-09-12008-s003.zip › control group stained with 0.1% Rhodamine B/37.png]

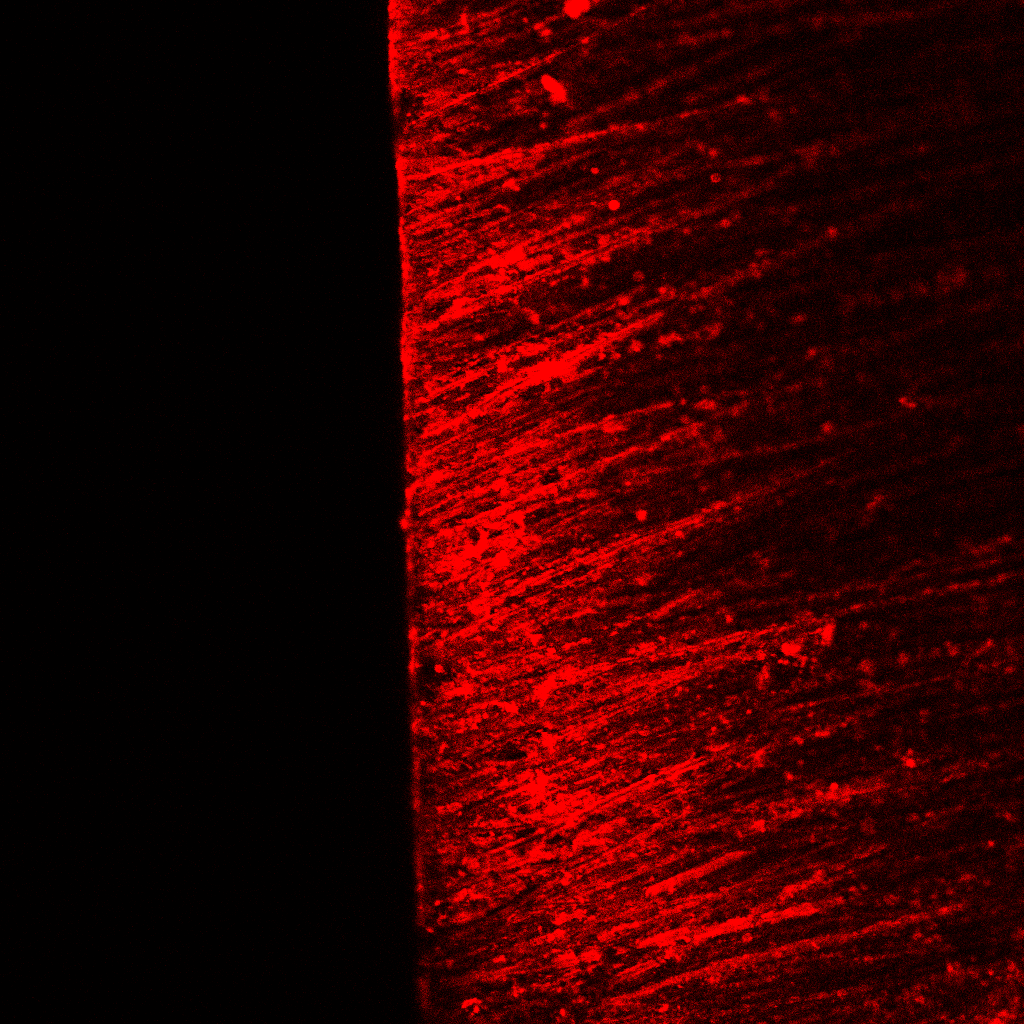

Supplement: Supplemental Information 3 [file peerj-09-12008-s003.zip › control group stained with 0.1% Rhodamine B/53.png]

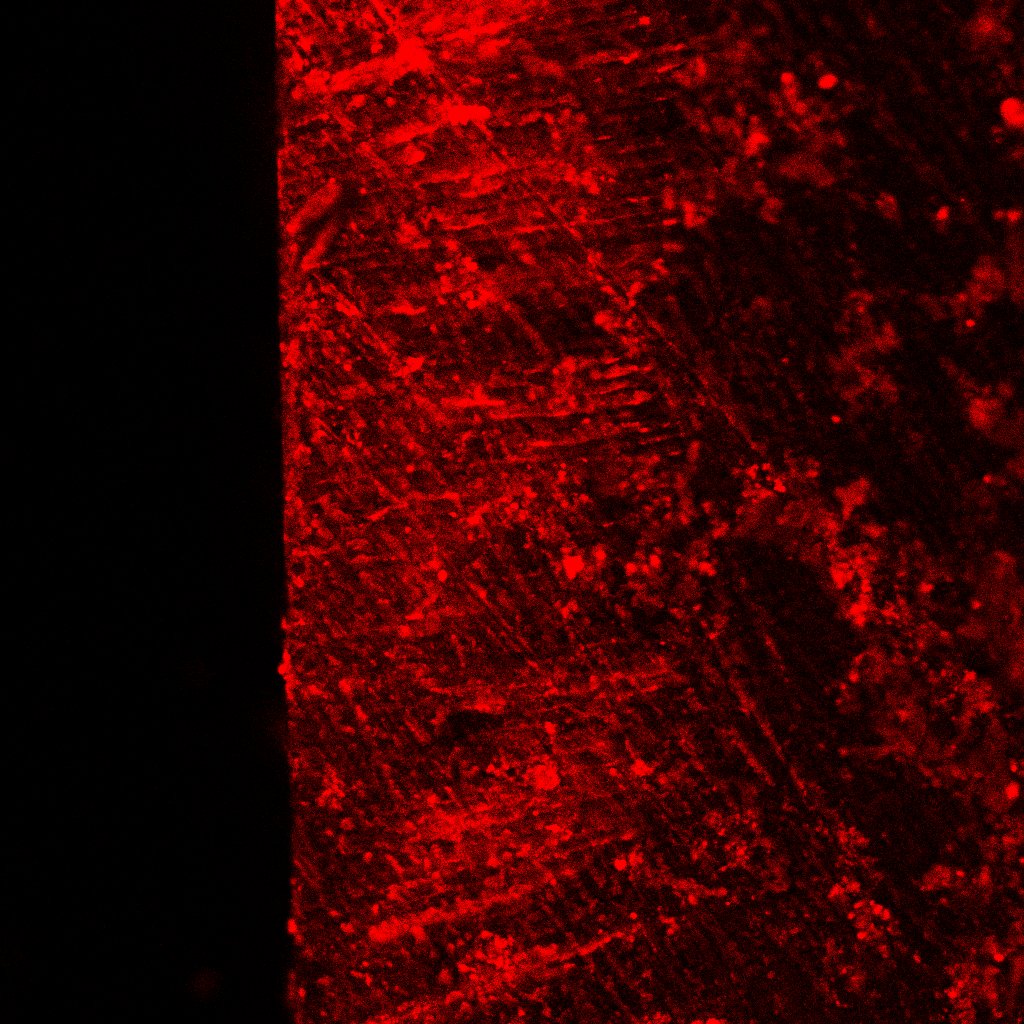

Supplement: Supplemental Information 3 [file peerj-09-12008-s003.zip › control group stained with 0.1% Rhodamine B/65.png]

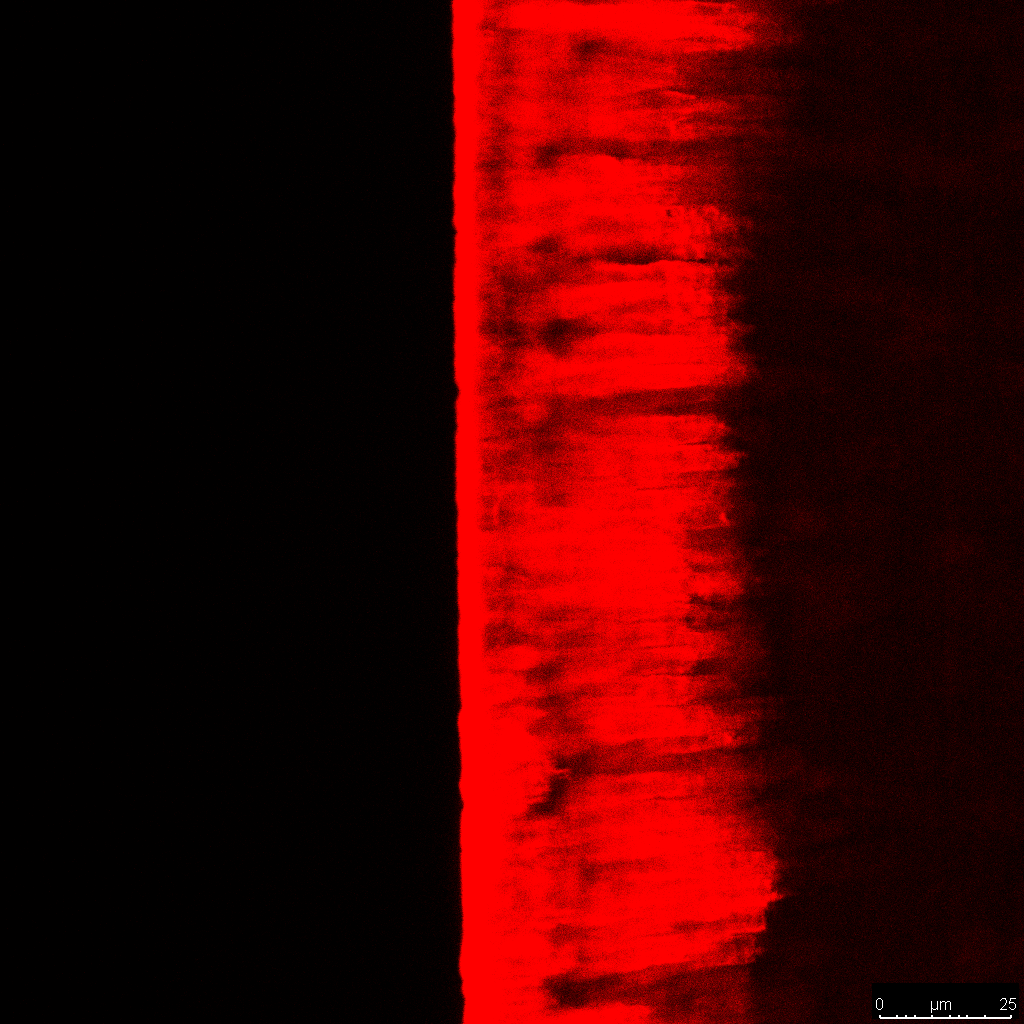

Supplement: Supplemental Information 3 [file peerj-09-12008-s003.zip › control group stained with 0.1% Rhodamine B/78.png]

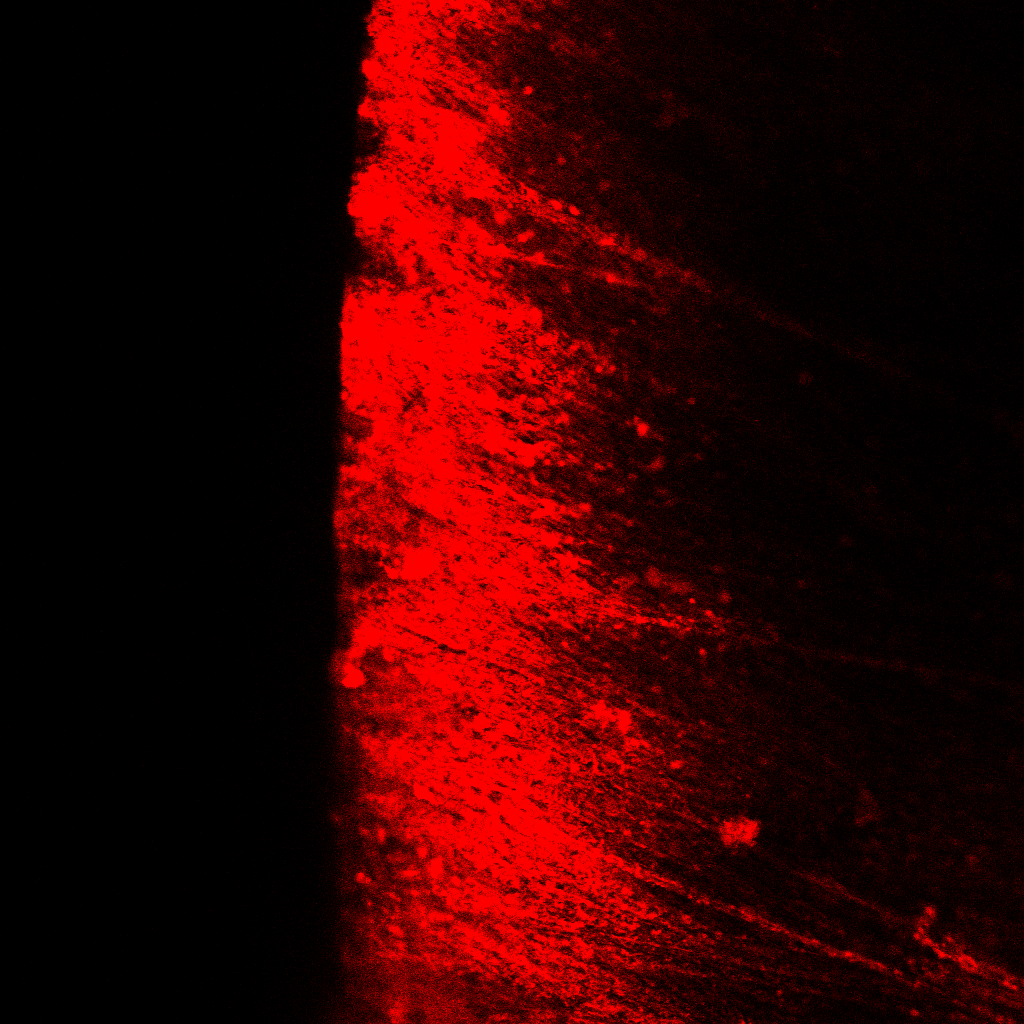

Supplement: Supplemental Information 3 [file peerj-09-12008-s003.zip › control group stained with 0.1% Rhodamine B/8.png]

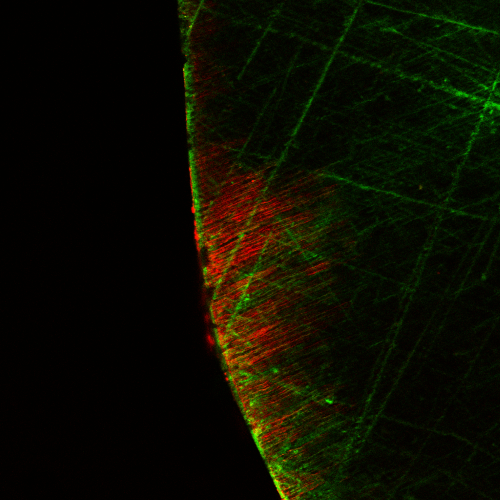

Supplement: Supplemental Information 4 [file peerj-09-12008-s004.zip › Double-fluorescence staining/1/Series002.png]

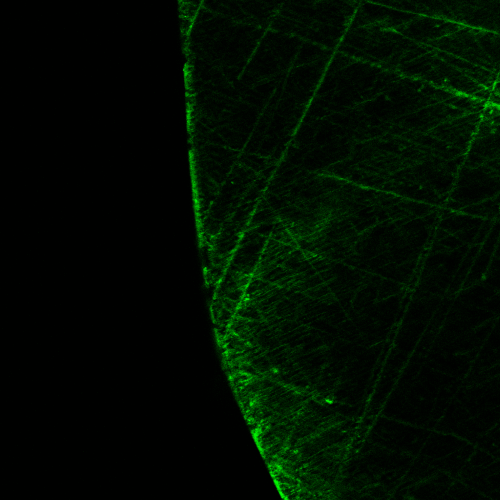

Supplement: Supplemental Information 4 [file peerj-09-12008-s004.zip › Double-fluorescence staining/1/Series002_ch00.png]

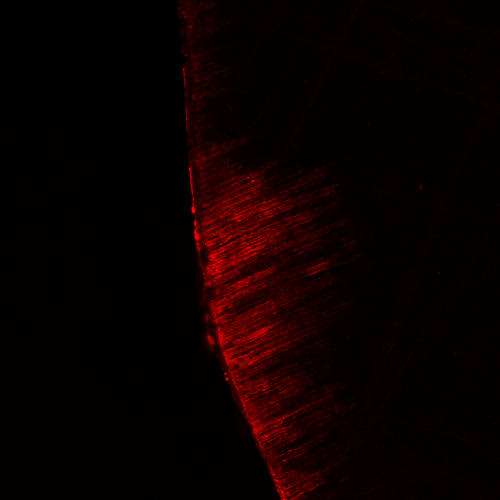

Supplement: Supplemental Information 4 [file peerj-09-12008-s004.zip › Double-fluorescence staining/1/Series002_ch01.png]

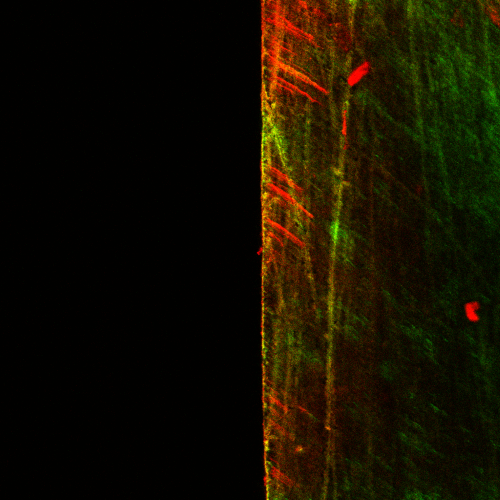

Supplement: Supplemental Information 4 [file peerj-09-12008-s004.zip › Double-fluorescence staining/10/Series001.png]

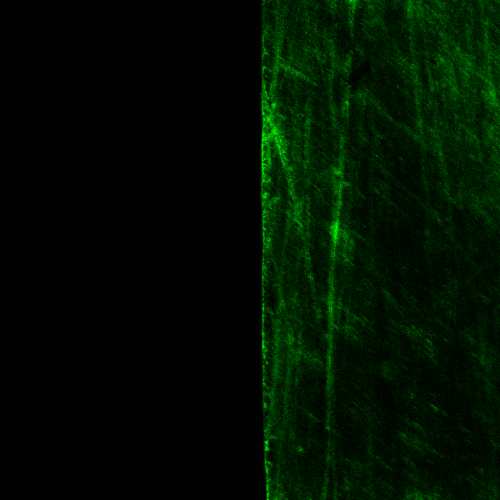

Supplement: Supplemental Information 4 [file peerj-09-12008-s004.zip › Double-fluorescence staining/10/Series001_ch00.png]

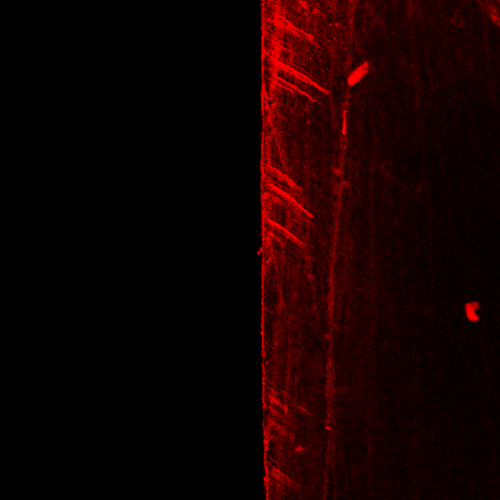

Supplement: Supplemental Information 4 [file peerj-09-12008-s004.zip › Double-fluorescence staining/10/Series001_ch01.png]

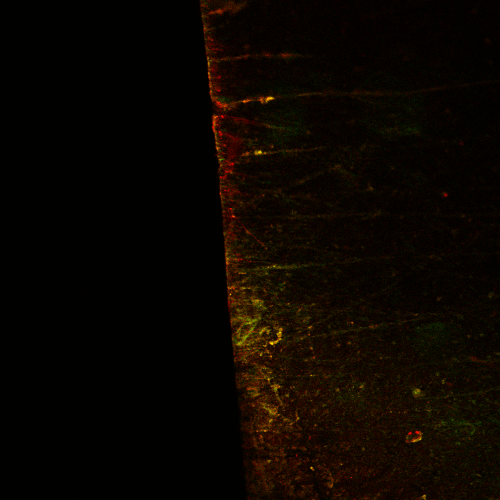

Supplement: Supplemental Information 4 [file peerj-09-12008-s004.zip › Double-fluorescence staining/2/Series001_z2.png]

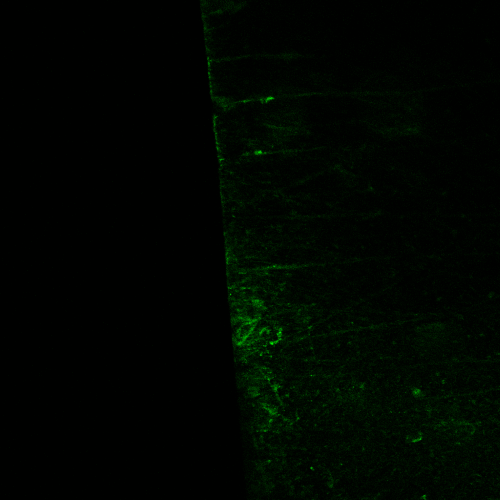

Supplement: Supplemental Information 4 [file peerj-09-12008-s004.zip › Double-fluorescence staining/2/Series001_z2_ch00.png]

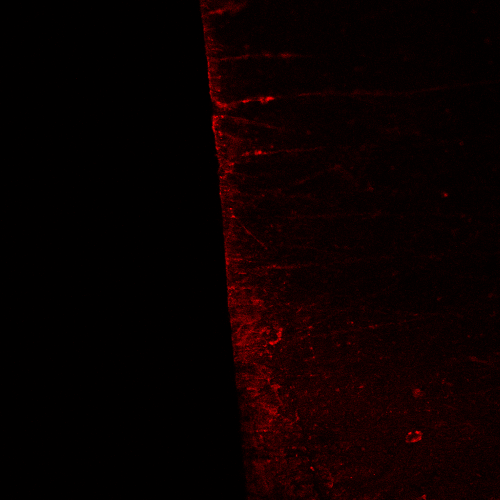

Supplement: Supplemental Information 4 [file peerj-09-12008-s004.zip › Double-fluorescence staining/2/Series001_z2_ch01.png]

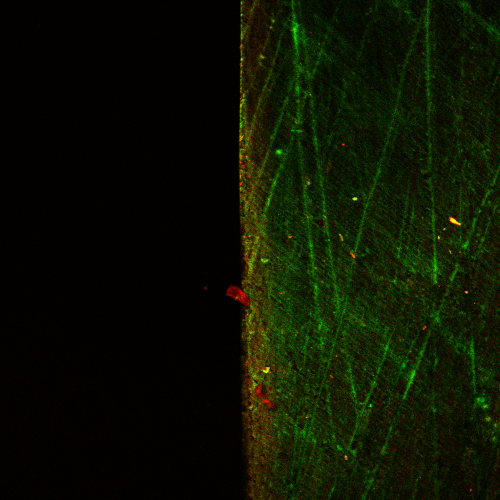

Supplement: Supplemental Information 4 [file peerj-09-12008-s004.zip › Double-fluorescence staining/3/Series001.png]

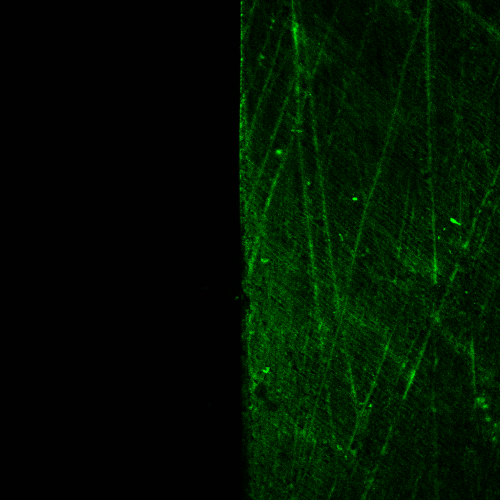

Supplement: Supplemental Information 4 [file peerj-09-12008-s004.zip › Double-fluorescence staining/3/Series001_ch00.png]

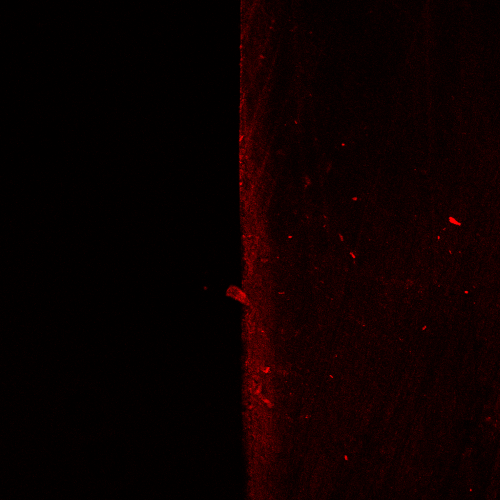

Supplement: Supplemental Information 4 [file peerj-09-12008-s004.zip › Double-fluorescence staining/3/Series001_ch01.png]

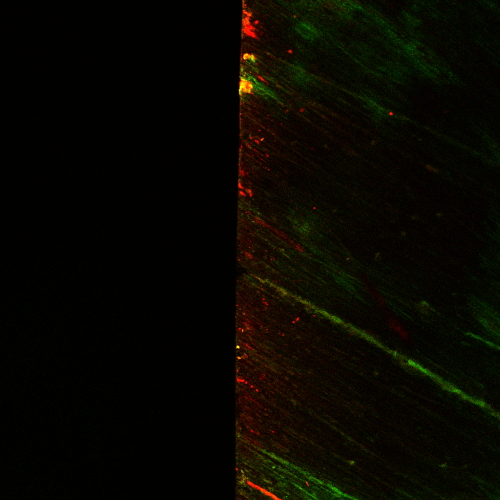

Supplement: Supplemental Information 4 [file peerj-09-12008-s004.zip › Double-fluorescence staining/4/Series001.png]

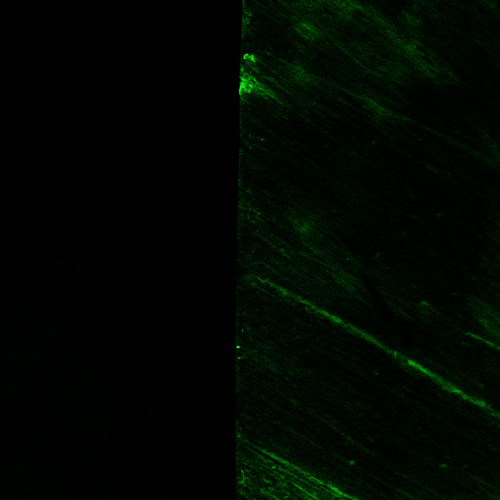

Supplement: Supplemental Information 4 [file peerj-09-12008-s004.zip › Double-fluorescence staining/4/Series001_ch00.png]

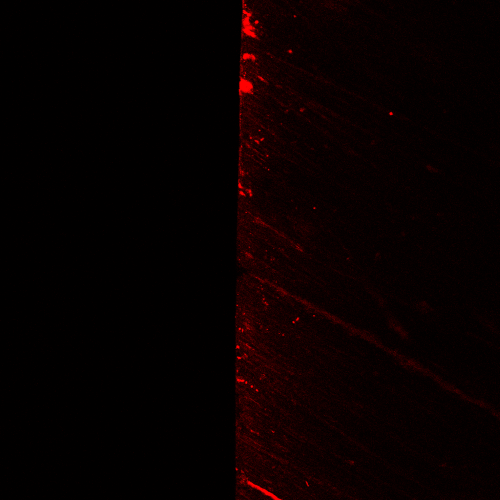

Supplement: Supplemental Information 4 [file peerj-09-12008-s004.zip › Double-fluorescence staining/4/Series001_ch01.png]

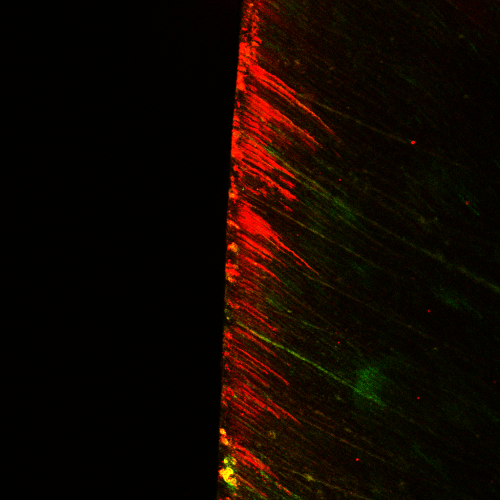

Supplement: Supplemental Information 4 [file peerj-09-12008-s004.zip › Double-fluorescence staining/5/Series003.png]

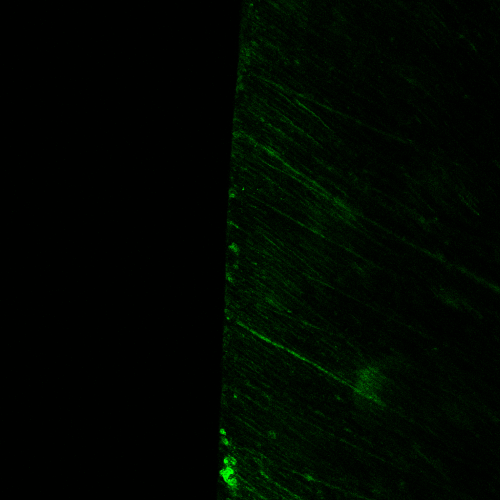

Supplement: Supplemental Information 4 [file peerj-09-12008-s004.zip › Double-fluorescence staining/5/Series003_ch00.png]

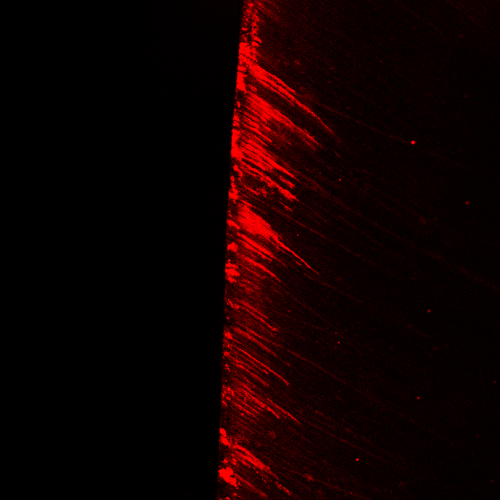

Supplement: Supplemental Information 4 [file peerj-09-12008-s004.zip › Double-fluorescence staining/5/Series003_ch01.png]

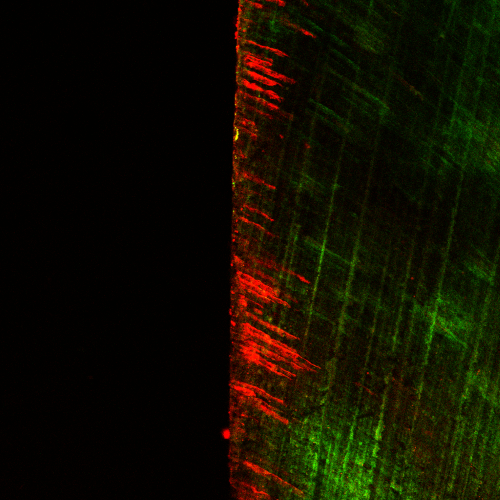

Supplement: Supplemental Information 4 [file peerj-09-12008-s004.zip › Double-fluorescence staining/6/Series001.png]

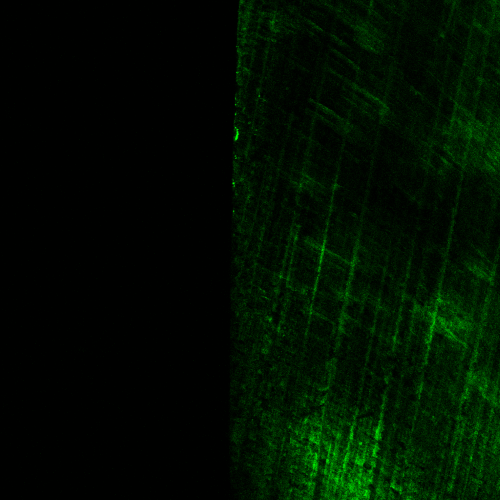

Supplement: Supplemental Information 4 [file peerj-09-12008-s004.zip › Double-fluorescence staining/6/Series001_ch00.png]

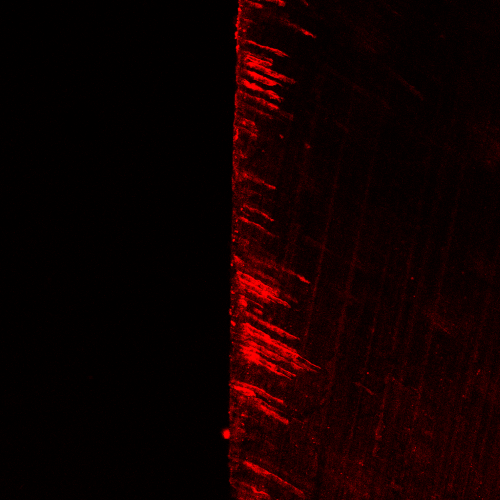

Supplement: Supplemental Information 4 [file peerj-09-12008-s004.zip › Double-fluorescence staining/6/Series001_ch01.png]

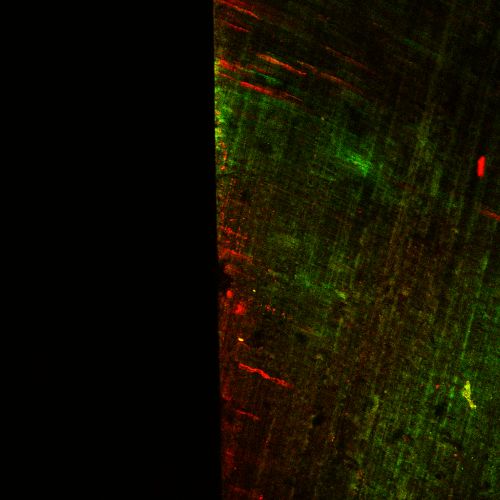

Supplement: Supplemental Information 4 [file peerj-09-12008-s004.zip › Double-fluorescence staining/7/Series003.png]

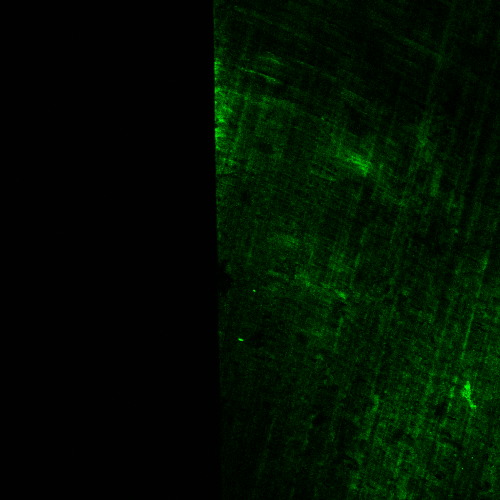

Supplement: Supplemental Information 4 [file peerj-09-12008-s004.zip › Double-fluorescence staining/7/Series003_ch00.png]

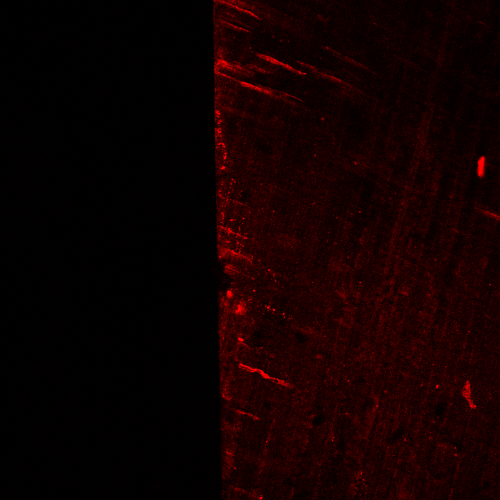

Supplement: Supplemental Information 4 [file peerj-09-12008-s004.zip › Double-fluorescence staining/7/Series003_ch01.png]

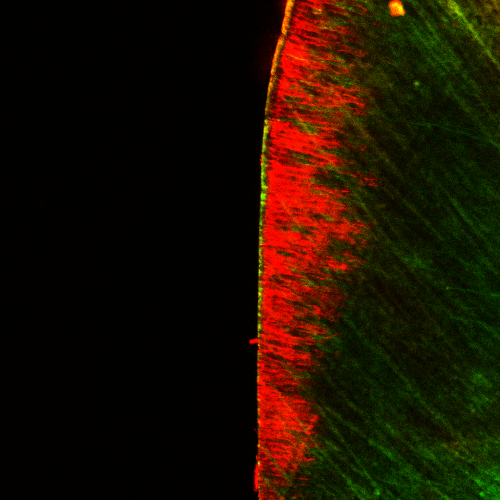

Supplement: Supplemental Information 4 [file peerj-09-12008-s004.zip › Double-fluorescence staining/8/Series001.png]

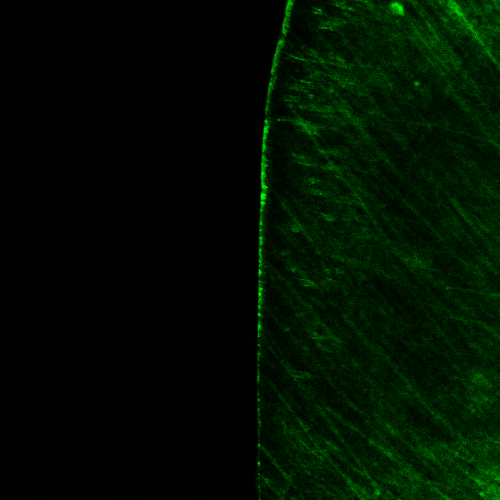

Supplement: Supplemental Information 4 [file peerj-09-12008-s004.zip › Double-fluorescence staining/8/Series001_ch00.png]

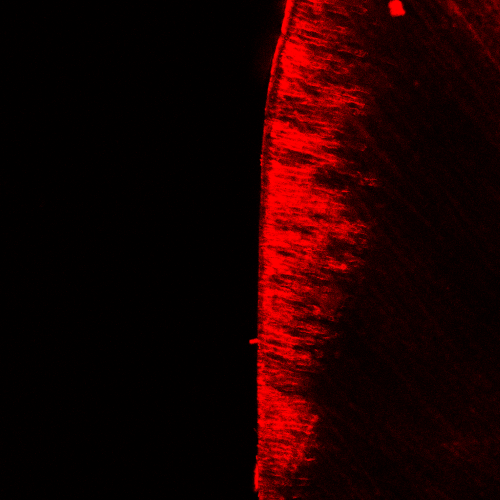

Supplement: Supplemental Information 4 [file peerj-09-12008-s004.zip › Double-fluorescence staining/8/Series001_ch01.png]

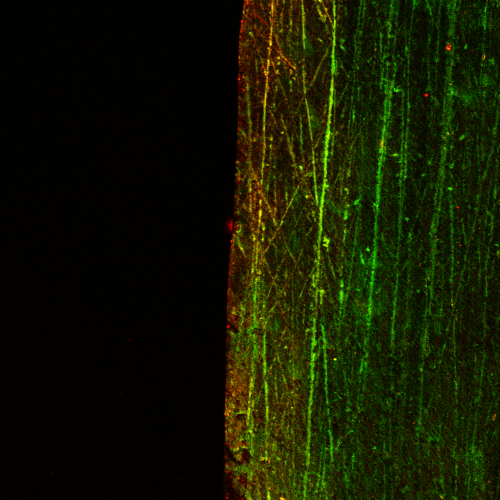

Supplement: Supplemental Information 4 [file peerj-09-12008-s004.zip › Double-fluorescence staining/9/Series003.png]

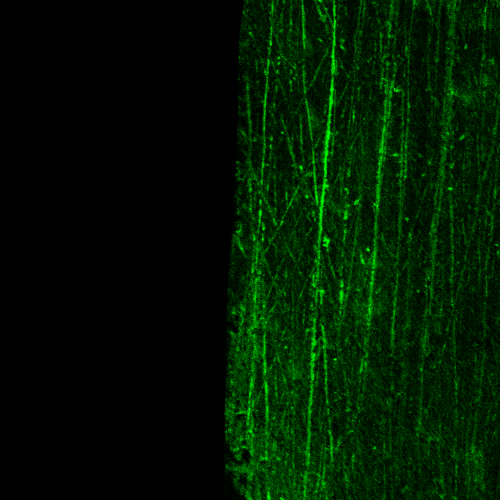

Supplement: Supplemental Information 4 [file peerj-09-12008-s004.zip › Double-fluorescence staining/9/Series003_ch00.png]

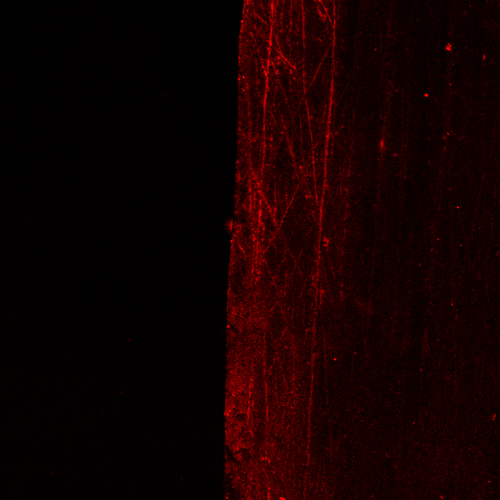

Supplement: Supplemental Information 4 [file peerj-09-12008-s004.zip › Double-fluorescence staining/9/Series003_ch01.png]
